# In Vitro Study of Antiviral Properties of Compounds Based on 1,4-Dioxane Derivative of Closo-Decaborate Anion with Amino Acid Ester Residues Against Influenza Virus A/IIV-Orenburg/83/2012(H1N1)pdm09

**DOI:** 10.3390/molecules29245886

**Published:** 2024-12-13

**Authors:** Timur M. Garaev, Ilya I. Yudin, Natalya V. Breslav, Tatyana V. Grebennikova, Evgenii Y. Matveev, Elizaveta A. Eshtukova-Shcheglova, Varvara V. Avdeeva, Konstantin Y. Zhizhin, Nikolay T. Kuznetsov

**Affiliations:** 1 Gamaleya National Research Center for Epidemiology and Microbiology, Ministry of Health of Russian Federation, Moscow 123098, Russia; tmgaraev@gmail.com (T.M.G.); yudinilya2000@yandex.ru (I.I.Y.); n.belyakova1983@gmail.com (N.V.B.); t_grebennikova@mail.ru (T.V.G.); 2 Kurnakov Institute of General and Inorganic Chemistry, Russian Academy of Sciences, Moscow 119991, Russia; cat1983@yandex.com (E.Y.M.); zhizhin@igic.ras.ru (K.Y.Z.); ntkuz@igic.ras.ru (N.T.K.); 3 Institute of Fine Chemical Technologies Named After M. V. Lomonosov, MIREA—Russian Technological University, Vernadskogo pr. 86, Moscow 119571, Russia; shchegs.ea@gmail.com

**Keywords:** boron clusters, cytotoxicity, antiviral activity, H1N1 virus

## Abstract

New derivatives of the *closo*-decaborate anion [B_10_H_9_–O(CH_2_)_2_O(CH_2_)_3_C(O)–L–OCH_3_]^2−^ (An) (**1**: L = Trp; **2**: L = His; **3**: L = Met; **4**: L = Ala(2-oxopyrrolidin-3-yl) (Pld) were synthesized and isolated as tetraphenylphosphonium salts (Ph_4_P)_2_An. Anions **1**^2−^; **2**^2−^; **3**^2−^, and **4**^2−^ contain a pendant functional group from the L-tryptophan methyl ester, L-histidine methyl ester, L-methionine methyl ester, or methyl 2-amino-3-(2-oxopyrrolidin-3-yl)propanoate (-Trp–OCH_3_, -His–OCH_3_, -Met–OCH_3_, or -Pld–OCH_3_) residue, respectively, bonded with the boron cluster anion through the oxybis[(ethane-2,1-diyl)oxy] spacer. This pacer is formed as a result of the nucleophilic opening of the attached dioxane molecule in the [B_10_H_9_O(CH_2_)_4_O]^−^ starting derivative. Sodium salts of the target compounds were isolated and used in biological experiments. It was established that among compounds Na_2_An (An = **1**–**4**), not all are capable of inhibiting the cytopathic effect of the virus in vitro. Sodium salts Na_2_An have a low toxic effect on a monolayer of continuous canine embryonic kidney (MDCK) cell line. Compounds Na_2_**1** and Na_2_**2** had IC_50_ of 5.0 and 20.0 μg/mL, respectively, while for compounds Na_2_**3** and Na_2_**4**, IC_50_ values could not be achieved at the concentrations studied. The studies performed for molecular docking of the anionic part of **1**^2−^ and **2**^2−^ with the transmembrane domain of viroporin M2 show some differences in the location of these two ligands inside the M2 canal pore.

## 1. Introduction

Influenza A viruses have accompanied mammals, including humans, for many centuries, causing seasonal epidemics and periodic pandemics with high morbidity and mortality rates. Due to the ability of influenza viruses to undergo reassortment on one hand and their zoonotic nature on the other, new strains with pandemic potential continue to emerge. Moreover, these newly arising strains can evade the therapeutic effects of existing anti-influenza drugs, as was the case with adamantane compounds (amantadine and rimantadine) more than 20 years ago and is currently happening with neuraminidase inhibitors (oseltamivir). Such threats from the evolution of influenza viruses underscore the urgent need for the development of new antiviral agents.

An important target for the therapy of infections caused by the influenza virus is the M2 proton-conducting channel in the viral particle’s envelope. It is hypothesized that M2 channel inhibitors are built in the following matter. They feature a hydrophobic fragment represented by an adamantane residue and a functional group responsible for forming non-covalent bonds with amino acid residues on the inner surface of the channel’s pore. It was shown that the adamantyl residue can be replaced by other hydrophobic groups, for example, by terpene derivatives, condensed aromatic systems, and other hydrocarbons [1,2].

In recent years, research on boron-based compounds as potential antiviral agents has gained momentum, given their unique chemical properties and biological activity. Recently, boron cluster and carborane-based compounds have increasingly been used in the development of new antiviral and antimicrobial agents [3,4,5,6,7,8,9,10,11]. The chemistry of carboranes is quite advanced; however, it should be noted that using carborane fragments in these studies does not guarantee success, specifically achieving compounds with high antiviral activity. For example, in a detailed study [12], the authors described the synthesis of a carborane analog of oseltamivir (Figure S1) and compared its biological activity with that of the reference prodrug and its active form—the carboxylic acid of oseltamivir. Oseltamivir is considered a prodrug that requires hydrolytic biotransformation (ester bond cleavage) to convert into its active form—a neuraminidase inhibitor [13]. Given the established role of boron clusters in medicinal chemistry as modulators of pharmacophore properties, the authors proposed replacing the ester moiety in oseltamivir with a carborane framework. However, further studies showed that the resulting carborane ester exhibited significantly lower in vitro antiviral activity than the reference drug. This suggests that the combination of oseltamivir with the carborane cluster led to a reduction in inhibitory activity.

Therefore, it seems quite reasonable to synthesize and investigate the antiviral activity of compounds containing homonuclear cluster fragments. Boron cluster anions are considered particularly promising as membrane carriers for the attachment of functionally active groups [14,15,16,17,18,19,20,21,22,23,24,25,26,27,28,29,30] due to their low toxicity, with a 50% cytotoxic concentration (CT_50_) exceeding 250.0 µg/mL [31]. Additionally, their ionic nature allows for the formation of salts with good solubility in water.

Previously, we proposed an influenza A virus replication inhibitor [B_10_H_9_–O(CH_2_)_4_C(O)–His–OCH_3_]^2−^ based on the residue of L-histidine methyl ester containing a boron cluster with a linker group [32] (Figure S2). The antiviral efficacy of Na_2_[B_10_H_9_–O(CH_2_)_4_C(O)–His–OCH_3_] was evaluated in vitro against the influenza virus A/Moscow/01/2009 (H1N1)pdm09. It was found that this compound exhibited antiviral activity at a concentration of 5.0 μg/mL and showed no cytotoxic effects at concentrations up to 160.0 μg/mL. Additionally, this compound was tested for its antiviral activity against the SARS-CoV-2 coronavirus in vitro [33]. Using Vero-E6 cell culture, it was found that the histidine derivative is able to suppress virus replication, exhibiting moderate antiviral activity against SARS-CoV-2. Given considerable effectiveness and low toxicity of synthetic derivatives of boron cluster compounds with amino acid residues, this field seems to be interesting for creating new antiviral drugs targeting current strains of influenza A.

The aim of this work was to modify the structure of the inhibitor of influenza A virus based on the boron cage to create new derivatives [B_10_H_9_–O(CH_2_)_2_O(CH_2_)_3_C(O)–L–OCH_3_]^2−^ (L = Trp, His, Met, or Pld), isolate the key compounds as human-friendly sodium salts, and evaluate their antiviral properties against an adamantane-resistant strain of influenza A virus. The choice of L-tryptophan and L-histidine as substituents is based on their known roles in modulating biological activity and their potential to enhance the antiviral efficacy of the boron cluster anion. In contrast to the aromatic heterocyclic substituents of L-histidine (imidazole) and L-tryptophan (indole), a saturated heterocycle (2-oxopyrrolidine) and an aliphatic sulfur-containing group of L-methionine were used.

## 2. Results and Discussion

New synthetic compounds with innovative molecular structures have the potential to significantly enhance the quality of treatment for patients and improve public health outcomes [34,35]. Innovative molecular structures are defined by chemical compositions that have not yet been approved for treating and preventing disease. This represents a crucial focus in the discovery and development of next-generation drugs with distinct therapeutic properties.

**Scheme 1:**
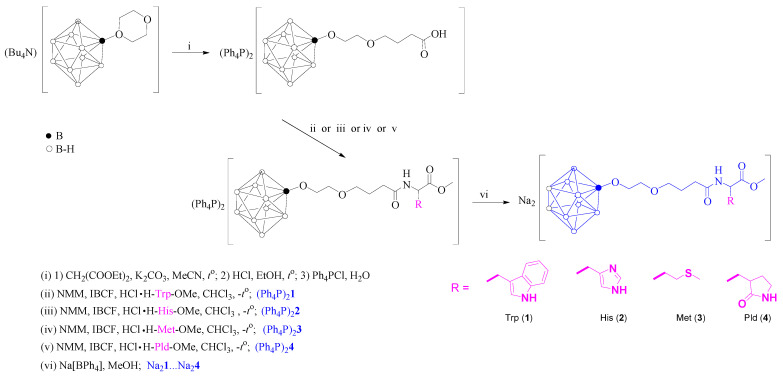
Synthesis of substituted *closo*-decaborates with L-amino acid methyl esters, where R represents a various side group of the L-amino acid: indole (for Trp) (anion **1**^2−^), imidazole (for His) (anion **2**^2−^), (methylsulfanyl)ethane (for Met) (anion **3**^2−^), or 2-oxopyrrolidin (for Pld) (anion **4**^2−^).

In this study, we synthesized new *closo*-decaborates with pendant histidine, tryptophan, methionine methyl ester residues, or methyl 2-amino-3-(2-oxopyrrolidin-3-yl)propanoate as functional groups. These groups are separated from the boron cage by an alkoxy spacer, created through the opening of the cyclic substituent (dioxane). The synthesis of these substituted derivatives was performed according to Scheme 1.

Scheme 1 shows the step-by-step synthesis of Na_2_[B_10_H_9_–O(CH_2_)_2_O(CH_2_)_3_C(O)–Trp–OCH_3_] (compound Na_2_**1**), Na_2_[B_10_H_9_–O(CH_2_)_2_O(CH_2_)_3_C(O)–His–OCH_3_] (compound Na_2_**2**), Na_2_[B_10_H_9_–O(CH_2_)_2_O(CH_2_)_3_C(O)–Met–OCH_3_] (compound Na_2_**3**), or Na_2_[B_10_H_9_–O(CH_2_)_2_O(CH_2_)_3_C(O)–Pld–OCH_3_] (compound Na_2_**4**). In the first stage, the nucleophilic opening of the cyclic oxonium substituent (1,4-dioxane) in the starting compound (Bu_4_N)[B_10_H_9_O(CH_2_)_4_O] was achieved using malonic ester in the presence of potassium carbonate (i1). The resulting acyclic derivative was then hydrolyzed in the acidic solution of 11% hydrochloric acid in ethanol, as indicated by index (i2), to produce the pendant carboxyl group. The addition of Ph_4_PCl (i3) allowed us to isolate compound (Ph_4_P)_2_[B_10_H_9_O(CH_2_)_2_O(CH_2_)_2_CH_2_COOH]. In the final stage of the synthesis indicated by indexes (ii–v) in the scheme, the target derivative of an L-amino acid ester was obtained using the mixed anhydride method, with isobutyl chloroformate and *N*-methylmorpholine serving as condensing reagents.

For further biological tests, the substituted derivatives were obtained in the form of sodium salts Na_2_An (An = **1**–**4**) as indicated (vi). Preparing hydrophobic substituted boron cluster anions as salts with alkali metal cations offers two key advantages. First, it enables the production of water-soluble forms of the target compounds, thereby increasing their availability for use. This is particularly important for in vitro screening, where the use of organic solvents could introduce cytotoxic effects that might interfere with results. Second, this approach eliminates the toxic effects associated with the organic cation (Ph_4_P^+^), allowing the toxicity of the anionic component of the compounds to be accurately assessed.

**Figure 1:**
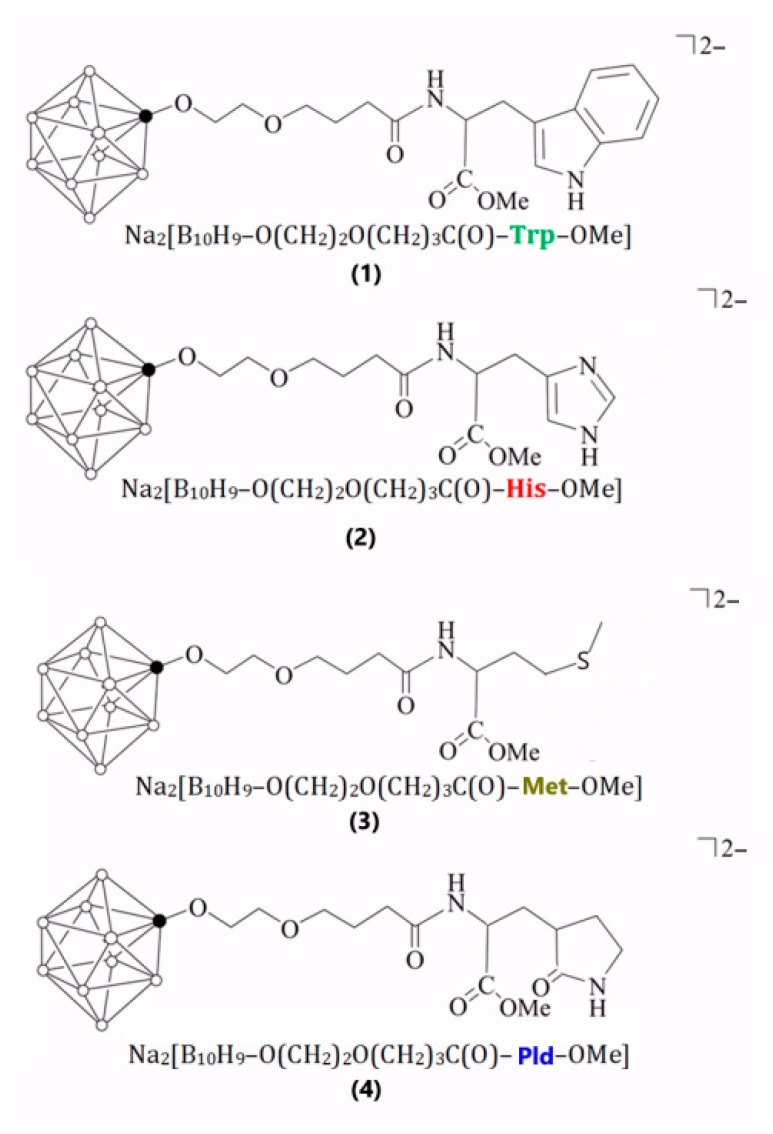
Structures of synthetic derivatives of the *closo*-decaborate anion with methyl esters of L-amino acids Na_2_**1**; Na_2_**2**; Na_2_**3**, and Na_2_**4**.

The anionic part of the resulting compounds has the following structural formula (Figure 1).

The compounds were identified by IR as well as ^1^H, ^11^B, and ^13^C NMR spectroscopies. The IR and multinuclear NMR spectroscopy data allow us to unambiguously judge the type of the attached pendant group via the spacer fragment of the opened dioxane cycle. In particular, the IR spectra of the products contain absorption bands of the B–H valence bonds from the boron cluster (at ~2450 cm^−1^, please see Supplementary Materials), as well as a number of bands characteristic of the amino acid component introduced. The ^11^B spectra allow us to conclude the opening of the exo-polyhedral 1,4-dioxane substituent, and the ^1^H and ^13^C spectra contain a complete set of signals from the alkoxy spacer chain and the pendant group. The whole set of spectral data indicates the formation of the desired functionalized *closo*-decaborates with the amino acid component introduced.

### 2.1. Determination of Cytotoxic Effect

The cytotoxicity of compounds Na_2_An was estimated on a monolayer of MDCK tissue culture cells; see Supplementary Materials for details.

**Figure 2:**
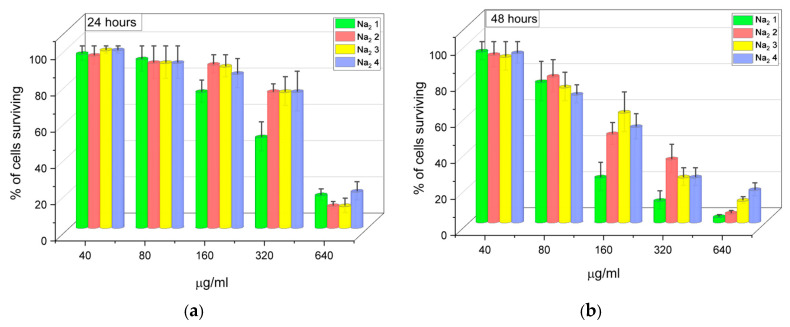
Toxicity of compounds Na_2_**1**–Na_2_**4** after (**a**) 24 h and (**b**) 48 h exposure to MDCK cell culture (*n* = 5).

The optical density (OD) of the solution in each well was measured at 490 nm (with a reference wavelength of 620 nm) using a Synergy HT tablet fluorimeter from BioTek Instruments (USA) (Figure 2). The concentration that reduced the OD value by 50% compared to the cell control was defined as the 50% cytotoxic dose (CT_50_).

All four compounds were found to be low toxic to the MDCK cell monolayer: CT_50_ values were 80.0 μg/mL for compound Na_2_**1** and 160.0 μg/mL for compounds Na_2_**2**, Na_2_**3,** and Na_2_**4.** The result was recorded 48 h after the introduction of Na_2_**1**–Na_2_**4** compounds into the monolayer of MDCK cells.

### 2.2. Antiviral Activity of Compounds Na_2_An

The antiviral activity of compounds Na_2_**1**–Na_2_**4** was evaluated using 96-well plates with a formed monolayer of MDCK tissue culture cells. Rimantadine was used as a control, and the synthetic compounds were added to the cell monolayer at concentrations of 5.0, 10.0, and 20.0 µg/mL simultaneously with the viral infection. Infection of the cell monolayer was carried out by adding four dilutions of the virus (at a working dose of 10–10,000 TCID_50_) to different rows of wells, taking into account a 2-fold dilution of the virus-containing suspension followed by the addition of MEM medium (with dissolved Na_2_**1**–Na_2_**4** or rimantadine) in a volume of 100 μL. The virus without the drug was applied to the control wells, and a double volume of MEM medium was added to the control wells of the cell monolayer. The synthetic compounds Na_2_**1**–Na_2_**4**, rimantadine hydrochloride, and virus-containing suspension applied to the plates with cell monolayer were kept in a thermostat at 37 °C in a flow of 5% CO_2_ for 24 ± 4 h. Then, the medium containing all the above-described components was removed from the plates, and the cell monolayer was fixed for 30 min with 80% acetone solution in phosphate-buffered saline (PBS) in a volume of 100 μL per well at +4 °C. The acetone was poured off and the fixed cell monolayer was thoroughly washed by filling 3 times and pouring out 300 μL per well with PBS solution with 0.05% TWEEN 20. In the plates washed in this way, the level of expression of viral antigens was studied in comparison with the values for the viral control by the ELISA method.

The ELISA assay was conducted following previously described methods [36,37]. Details can be seen in Supplementary Materials.

**Figure 3:**
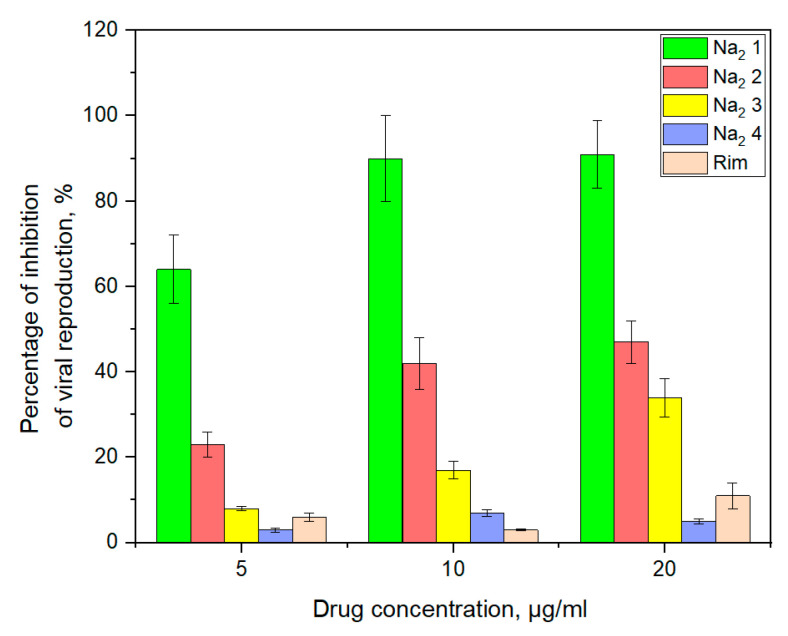
Inhibition of reproduction of pandemic strain influenza virus A/IIV-Orenburg/83/2012(H1N1)pdm09 under the action of synthetic compound Na_2_**1**–Na_2_**4** and rimantadine (Rim) in MDCK cell culture.

Figure 3 presents the effect of three different concentrations of Na_2_**1**–Na_2_**4** on the replication of the influenza A virus under discussion. Compound Na_2_**1** was found to effectively inhibit the influenza A(H1N1)pdm09 virus strain resistant to adamantane-based drugs with a 50% inhibitory dose (IC_50_) of 5.0 μg/mL. In contrast, compound Na_2_**2** containing L-histidine residue achieved IC_50_ in vitro experiments only at 20.0 μg/mL when the average activity was about 50%. Compound Na_2_**3** demonstrated a low effect of protecting the cell monolayer from the cytopathic action of the virus; the activity at 20.0 μg/mL was only 34%. Compound Na_2_**4** did not show antiviral activity in vitro at a concentration of 20.0 μg/mL. In addition, rimantadine hydrochloride, which was used as a standard for determining resistance to adamantane-based drugs, was also ineffective, indicating that the influenza virus strain is resistant to both rimantadine and amantadine due to their cross-resistance.

Based on the data presented in Figure 2 and Figure 3, the selectivity index (Si = CT_50_/IC_50_) for compound Na_2_**1** was 16. Despite the fact that compound Na_2_**2** is somewhat less toxic to the MDCK cell monolayer compared to its tryptophan analog, the selectivity index of Na_2_**2** is slightly less than 8 due to the fact that at a load of 20 μg/mL, the percentage of viral reproduction suppression was 47%, and CT_50_ was 160 μg/mL. It was not possible to calculate Si for compounds Na_2_**3** and Na_2_**4** because of the lack of IC_50_ antiviral activity at the concentrations studied.

The mechanism of action of compound Na_2_**1** remains unclear. However, it is conceivable that this compound could be developed into a new antiviral drug targeting the M2 ion-selective channel of the influenza A virus. It could potentially be used as a standalone treatment or as part of combination therapy, similar to the previously described analog Na_2_[B_10_H_9_–O(CH_2_)_4_C(O)–His–OCH_3_] [32].

The significant difference in antiviral potency between the four structurally similar compounds is not fully understood. The significantly reduced antiviral activity for the imidazole-containing derivative (Na_2_**2**) compared to the indole-containing derivative (Na_2_**1**) may be related to their molecular structures. The indole-containing anion **1**^2−^ demonstrated complete inhibition of viral replication in vitro at 10.0 and 20.0 μg/mL. To further study the effect of amino acid side groups on antiviral properties, it is planned to synthesize a series of compounds with different amino acid residues in the future. This will allow us to compare their biological properties and study the structure–activity relationship.

For the development of direct-acting drugs in modern bioorganic and pharmaceutical chemistry, it is crucial to predict the structure of a small inhibitor molecule (ligand) with a specific functional center of the target protein. The use of modern artificial intelligence methods for molecular docking algorithms allows us to reduce the time spent on searching for the optimal structure and understanding the mechanism of action of the inhibitor on the target protein [38].

In this work, the DIFFDOCK online service was used to perform the docking of the Na_2_**1** and Na_2_**2** leader compounds with the proton channel formed by four subunits of the M2 protein of the influenza A virus [39]. An important feature of this docking algorithm is the docking solution using a diffusion generative model (DGM) in the pose space of ligands for molecular docking. The docking process in this case is a set of diffusion states over the degrees of freedom involved in docking, such as the position of the ligand relative to the protein (location of the binding pocket), its orientation in the pocket, and torsion angles describing its conformation [40].

To reliably block the function of the M2 proton channel of the influenza A virus, located in the outer membrane of the virus, it is necessary to block the ability of this protein to pump hydrogen ions into the virion membrane after entering the endosome. This process is associated with the unpacking of the virus and the beginning of the replication cycle. The amino acid residues His37 and Trp41, turned with their functional groups (imidazole and indole) inside the channel pore, participate in the process of proton transfer. This pair, “His37–Trp41”, is a gateway mechanism in which the His37 residues serve as a hydrogen ion pump, and Trp41 serves as a gate for opening the M2 channel and allowing H^+^ to enter. The structure of the transmembrane region of the M2 channel is shown in Figure S3.

**Figure 4:**
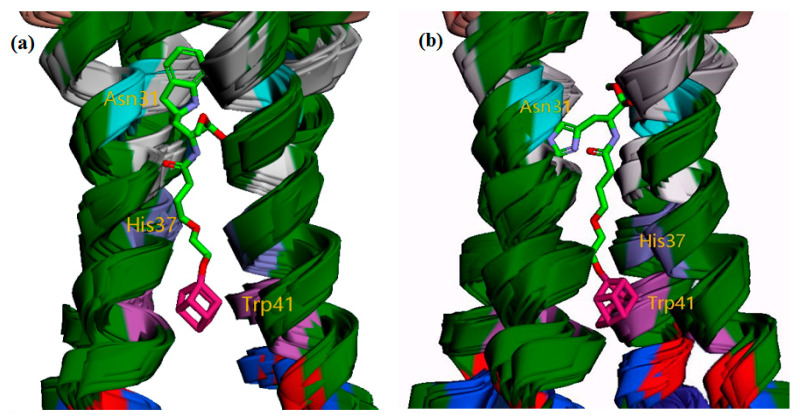
Complex of the crystallographic structure of the M2 channel of the influenza A virus (2KIH) and the synthetic blocker of ion channel function (**a**) Na_2_**1** and (**b**) Na_2_**2**. The fundamentally important amino acid residues of the transmembrane domain of the M2 channel are indicated.

As a result of the molecular modeling of the docking of the M2 protein pore and compound Na_2_**1**, the most preferable solution was to locate the ligand in the channel pore, when the borohydride cluster is located in the region of residues that form a pair of the channel’s gateway mechanism, “His37–Trp41”, and the tryptophan methyl ester residue is located in the region of the marker substitution of the rimantadine-resistant strain, i.e., Asn31. According to our previously put forward concept regarding adamantane-substituted amino acids in which the adamantane carbocycle acts as a membranotropic carrier for the functional group of the amino acid residue [31,34,35], the role of the carrier is determined by the boron cluster. Therefore, it is located lower in the channel pore than the functional group of the tryptophan residue (Figure 4a). The location of the **2**^2−^ anion in the M2 channel pore, namely, in its transmembrane region, is very similar to that found for the **1**^2−^ anion, but there is a rotation of the imidazole ring of the terminal histidine residue toward the inside of the pore, compared to the location of the indole group of the tryptophan residue for compound **1**^2−^ (Figure 4b). It is quite difficult to assess the effect of such conformational differences on the proton conductivity of the M2 channel in the present in silico study; this requires additional docking calculations taking into account the factors of molecular dynamics (MD) simulation. However, the obtained results of in vitro activity indicate that the nature of the amino acid substituent probably significantly affects the proton conductivity of the M2 channel of influenza A virus.

## 3. Experiments

Reagents. Commercially available potassium carbonate (reagent grade), hydrochloric acid (36%, chemical grade) from “Khimmed” (Moscow, Russia), decaborane(14) (B_10_H_14_, 98%, Acros Organics, Geel, Belgium), L-tryptophan methyl ester hydrochloride (98%, Aldrich, St. Louis, MO, USA), L-histidine methyl ester dihydrochloride (97%, Aldrich, St. Louis, MO, USA), L-methionine methyl ester hydrochloride (97%, Aldrich, St. Louis, MO, USA). (S)-Methyl 2-amino)-3-((S)-2 oxopyrrolidin-3-yl)propanoate (H-DL-Ala(2-oxopyrrolidin-3-yl)-OMe (98%, CAS 1822551-21-2, Henan Tianfu Chemical Co., Ltd., Zhengzhou, China), *N*-methylmorpholine (NMM, Denver, CO, USA), and *iso*-butyl chloroformate (IBCF), malonic ester (98%, Aldrich, St. Louis, MO, USA), acetonitrile (99%, Aldrich, St. Louis, MO, USA), potassium carbonate (chemically pure, Khimmed, Moscow, Russia), hydrochloric acid (36%, chemically pure, Khimmed, Moscow, Russia), tetraphenylphosphonium chloride (99%, Aldrich, St. Louis, MO, USA), and sodium tetraphenylborate (99.5%, Aldrich, St. Louis, MO, USA) were used without further purification.

Tetrabutylammonium [2-(1-(1,4-dioxanium))]nonahydro-*closo*-decaborate (*n*-Bu_4_N)[B_10_H_9_O(CH_2_)_4_O] was synthesized starting with decaborane(14) according to a previously developed procedure [41]; 1,4-dioxane was purified according to the procedure reported in [42].

All solvents were preliminarily purified and distilled using standard methods. The completeness of the reactions was monitored using ^11^B NMR spectroscopy (a Bruker DPX-300 NMR spectrometer, Bruker, Rheinstetten, Germany) and infrared spectroscopy (an INFRALUM FT-02 Fourier transform IR spectrometer, St. Petersburg, Russia).

Methods. The compounds were identified by IR as well as ^1^H, ^11^B, and ^13^C NMR spectroscopies on the above mentioned devices. Details can be seen in Supplementary Materials.

Virological studies were carried out using the pandemic strain of influenza virus A/IIV-Orenburg/83/2012(H1N1)pdm09. We used the pandemic strain of the influenza virus A/IIV-Orenburg/83/2012(H1N1)pdm09, in the MDCK cell culture at the D.I. Ivanovsky Research Institute of Virology of the Ministry of Health of the Russian Federation in 2011–2012. The strain was resistant to the action of the drugs rimantadine and amantadine.

The study of the infectious titer of the influenza virus was carried out on the continuous cell line MDCK (NBL-2) (CCL-34, American Type Culture Collection, ATCC, Manassas, VA, USA). We used a 3-day monolayer of continuous MDCK cell line grown in Minimum Essential Medium (MEM). All procedures were performed on MEM medium with the addition of trypsin (TPCK treated, Sigma, Livonia, MI, USA) at a concentration of 2 μg/mL. The panels were incubated for 24 h at 37 °C, and then the reaction was stopped by fixing the cells with 80% acetone in phosphate buffer. The cellular enzyme-linked immunosorbent assay (ELISA) method was performed according to the method described previously [34,35].

Syntheses. (a) Tetraphenylphosphonium 2-[2-(2-(2-methylcarboxy)ethoxy)ethoxy]nonahydro-*closo*-decaborate (Ph_4_P)_2_[B_10_H_9_O(CH_2_)_2_O(CH_2_)_2_CH_2_COOH]. (Bu_4_N)[B_10_H_9_O_2_C_4_H_8_] (1.00 g, 2.23 mmol), malonic ester (1.02 mL, 6.9 mmol), and potassium carbonate (1.54 g, 11.2 mmol) were added to 50.0 mL of acetonitrile, and the resulting suspension was boiled with stirring for 2 h. After cooling, the resulting system was filtered from excess unreacted potassium carbonate. The resulting light yellow filtrate was evaporated almost completely to a small amount of yellow viscous liquid. A solution of 70.0 mL of 11% hydrochloric acid solution and 15.0 mL of ethanol was added to it. The resulting solution was heated at boiling temperature for 24 h. Next, the cooled clear solution was evaporated to dryness, the resulting white powder was dissolved in water (10 mL), and a solution of Ph_4_PCl (1.67 g, 4.46 mmol) in water (15 mL) was added. The yellow precipitate formed was filtered off and dried in a high vacuum (yield, 1.70 g (81%)). 

The results were as follows: ^1^H NMR (DMSO-d_6_, δ, ppm): −1.00−1.00 (m, 9H), 1.73–1.83 (m, 2H), 2.15 (t, *J* = 7.1 Hz, 2H), 3.20 (t, *J* = 7.4 Hz, 2H), 3.36–3.50 (m, 4H), and 12.2 (br. s, 1H); ^11^B {^1^H} NMR (DMSO-d_6_, δ, ppm): −30.8, −27.8, −21.2, −4.3, and −0.5; and ^13^C NMR (DMSO-d_6_, δ, ppm): 28.3, 33.2, 51.8, 69.2, 70.4, 71.7, and 169.4.

(b) Synthesis of (Ph_4_P)_2_[B_10_H_9_O(CH_2_)_2_O(CH_2_)_2_CH_2_C(O)-Trp-OCH_3_] (Ph_4_P)_2_**1**. A solution of compound (Ph_4_P)_2_[B_10_H_9_O(CH_2_)_2_O(CH_2_)_2_CH_2_COOH] (0.35 g, 0.37 mmol) was prepared in 10.0 mL and 0.034 mL (0.37 mmol) of N-methylmorpholine (NMM) was added. It was cooled to −25 °C and, with stirring, 0.052 mL (0.37 mmol) of iso-butyl chloroformate (IBCF) was added to the reaction mass. The reaction mixture was stirred for 10 min. Then, a solution of 0.10 g (0.39 mmol) of tryptophan methyl ester hydrochloride in 10.0 mL CHCl_3_ with 0.036 mL (0.39 mmol) NMM previously prepared and cooled to −20 °C was added. The reaction mixture was stirred for 30 min at −15 °C, then for another 1 h at 0 °C and 10 h at room temperature. The reaction mass was transferred to a separatory funnel and washed successively (H_2_O (10.0 mL × 1); 0.5 N sulfuric acid (4.0 mL × 1); 0.5 N KHCO_3_ (10.0 mL × 2)), then acidified with 1 N H_2_SO_4_ and washed with H_2_O (5.0 mL × 1). The organic layer was separated and dried with anhydrous Na_2_SO_4_. Chloroform was removed in vacuum on a rotary evaporator (Chongqing Drawell Instrument Co., Ltd., Chongqing, China) at 45 °C and 15 mmHg, resulting in foamed oil, which solidified after some time (yield, 0.33 g (78.5%); solid white powder).

The following results were obtained: ^1^H NMR (DMSO-d_6_, δ, ppm): −1.00–1.03 (m, 9H), 1.73–1.85 (m, 2H), 2.17 (t, *J* = 6.9 Hz, 2H), 3.13–3.49 (m, 8H), 3.62 (s, 3H), 4.21 (t, *J* = 6.3 Hz, 1H), 6.51 (br. s, 1H), 6.99 (dd, *J* = 8.6, 7.4 Hz, 1H), 7.05 (dd, *J* = 8.9, 7.4 Hz, 1H), 7.26 (s, 1H), 7.36 (d, *J* = 8.6 Hz, 1H), 7.49 (d, *J* = 8.9 Hz, 1H), 7.70–8.00 (m, 40H), and 11.07 (s, 1H); ^11^B {^1^H} NMR (DMSO-d_6_, δ, ppm): −31.7, −26.9, −21.4, −3.3, and −0.6; ^13^C NMR (DMSO-d_6_, δ, ppm): 26.4, 28.9, 32.8, 52.4, 52.9, 68.3, 69.6, 72.0, 106.6, 111.5, 117.9, 118.5, 121.0, 124.8, 126.9, 130.3, 130.5, 134.4, 134.5, 135.3, 136.1, 170.1, and 173.7; and IR (KBr, cm^−1^): 2447 (ν(B-H)) and 1749 ((ν(C=O)).

(c) Synthesis of sodium methyl 2-[4-(2-methoxyethoxy)butanamido]-3-(1H-indol-3-yl)propanoate-nonahydro-*closo*-decaborate Na_2_[B_10_H_9_O(CH_2_)_2_O(CH_2_)_3_C(O)-Trp-OCH_3_] Na_2_**1**. Compound Na_2_**1** was obtained by a methatesis reaction with sodium tetraphenylborate. (Ph_4_P)_2_**1** (0.22 g, 0.19 mmol) was dissolved in 2.0 mL methanol. A solution of Na[BPh_4_] (0.13 g, 0.38 mmol) in 2.0 mL of methanol was added, and the formation of a white precipitate was observed. The precipitate was filtered off, the solution was evaporated to obtain a white hygroscopic powder, which was dried in high vacuum (yield, 0.044 g (47%)).

The following results were obtained: ^1^H NMR (DMSO-d_6_, δ, ppm): −1.00–1.03 (m, 9H), 1.73–1.85 (m, 2H), 2.17 (t, *J* = 6.9 Hz, 2H), 3.13–3.49 (m, 8H), 3.62 (s, 3H), 4.21 (t, *J* = 6.3 Hz, 1H), 6.99 (dd, *J* = 8.6, 7.4 Hz, 1H), 7.05 (dd, *J* = 8.9, 7.4 Hz, 1H), 7.26 (s, 1H), 7.36 (d, *J* = 8.6 Hz, 1H), 7.49 (d, *J* = 8.9 Hz, 1H), 8.95 (s, 1H), and 11.07 (s, 1H); and ^13^C NMR (DMSO-d_6_, δ, ppm): 26.4, 28.9, 32.8, 52.4, 52.9, 68.3, 69.6, 72.0, 106.6, 111.5, 117.9, 118.5, 121.0, 124.8, 126.9, 136.1, 170.1, and 173.7.

(d) Compound (Ph_4_P)_2_**2** was prepared according to a procedure similar to that used to prepare compound (Ph_4_P)_2_**1** using 0.35 g (0.37 mmol) of (Ph_4_P)_2_[B_10_H_9_O(CH_2_)_2_O(CH_2_)_2_CH_2_COOH] and 0.1 g (0.37 mmol) of another aminoacid 2HCl · H-His-OCH_3_.

The following results were obtained: ^1^H NMR (DMSO-d_6_, δ, ppm): −0.75–1.00 (m, 9H), 1.71–1.79 (m, 2H), 2.15 (t, *J* = 7.2 Hz, 2H), 3.27–3.44 (m, 8H), 3.73 (s, 3H), 4.44 (t, *J* = 6.9 Hz, 1H), 6.50 (br. s, 1H), 7.44 (s, 1H), 7.71–8.01 (m, 40H), 8.80 (s, 1H), and 11.66 (br. s, 1H); ^11^B {^1^H} NMR (DMSO-d_6_, δ, ppm): −31.5, −27.0, −21.3, −3.4, and −0.4; ^13^C NMR (DMSO-d_6_, δ, ppm): 25.4, 32.4, 33.2, 51.3, 52.9, 60.1, 69.7, 72.0, 117.8, 127.3, 130.3, 130.5, 134.4, 134.6, 135.3, 168.6, and 173.8; and IR (KBr, cm^−1^): 2448 (ν(B-H)) and 1761 ((ν(C=O)).

(e) Synthesis of sodium methyl 2-[4-(2-methoxyethoxy)butanamido]-3-(1H-imidazole-5-yl)propanoate-nonahydro-*closo*-decaborate Na_2_[B_10_H_9_O(CH_2_)_2_O(CH_2_)_2_CH_2_COHis] Na_2_**2**. (Ph_4_P)_2_**2** (0.220 g, 0.20 mmol) was dissolved in 2 mL of methanol. A solution of Na[BPh_4_] (0.137 g, 0.40 mmol) in 2 mL of methanol was added to this solution, and a white precipitate formed. The precipitate was filtered, and the solution was evaporated to obtain a white hygroscopic powder, which was dried in high vacuum (yield, 0.045 g (49%)).

The following results were obtained: ^1^H NMR (DMSO-d_6_, δ, ppm): −0.75–1.00 (m, 9H), 1.71–1.79 (m, 2H), 2.15 (t, *J* = 7.2 Hz, 2H), 3.27–3.44 (m, 8H), 3.73 (s, 3H), 4.51 (td, *J* = 7.3, 5.7 Hz, 1H), 7.45 (s, 1H), 8.46 (d, *J* = 5.7 Hz, 2H), and 8.78 (s, 1H); and ^13^C NMR (DMSO-d_6_, δ, ppm): 25.4, 32.4, 33.2, 51.3, 52.9, 60.1, 69.7, 72.0, 117.8, 127.3, 133.3, 173.8, and 175.4.

(f) Compound (Ph_4_P)_2_**3** was prepared according to a procedure similar to that used to prepare compound (Ph_4_P)_2_**1** using 0.25 g (0.27 mmol) of (Ph_4_P)_2_[B_10_H_9_O(CH_2_)_2_O(CH_2_)_2_CH_2_COOH] and 0.053 g (0.27 mmol) of another aminoacid HCl · H-Met-OCH_3_.

The following results were obtained: ^1^H NMR (DMSO-d_6_, δ, ppm): −0.99–0.99 (m, 9H), 1.73–1.85 (m, 2H), 2.05–2.12 (m, 7H), 2.54–2.69 (m, 2H), 3.31–3.47 (m, 8H), 3.75 (s, 3H), 4.13 (t, *J* = 6.8 Hz, 1H), 6.50 (br. s, 1H), and 7.71–8.01 (m, 40H); ^11^B {^1^H} NMR (DMSO-d_6_, δ, ppm): −31.4, −27.4, −21.5, −3.8, and −0.1; ^13^C NMR (DMSO-d_6_, δ, ppm): 14.2, 24.2, 28.3, 29.6, 33.4, 51.0, 52.7, 68.1, 69.6, 71.9, 130.3, 130.5, 134.4, 134.6, 135.3, 169.7, and 171.7; and IR (KBr, cm^−1^): 2445 (ν(B-H)) and 1746 ((ν(C=O)).

(g) Synthesis of sodium methyl 2-[4-(2-methoxyethoxy)butanamido]-3-(2-amino-4-(methylsulfanyl)butanoate-nonahydro-*closo*-decaborate Na_2_[B_10_H_9_O(CH_2_)_2_O(CH_2_)_2_CH_2_COMet] Na_2_**3**. (Ph_4_P)_2_**3** (0.218 g, 0.20 mmol) was dissolved in 2 mL of methanol. A solution of Na[BPh_4_] (0.137 g, 0.40 mmol) in 2 mL of methanol was added to this solution, and a white precipitate formed. The precipitate was filtered, and the solution was evaporated to obtain a white hygroscopic powder, which was dried in high vacuum (yield, 0.040 g (44%)).

The following results were obtained: ^1^H NMR (DMSO-d_6_, δ, ppm): −0.99–0.99 (m, 9H), 1.73–1.85 (m, 2H), 2.05–2.12 (m, 7H), 2.54–2.69 (m, 2H), 3.31–3.47 (m, 8H), 3.75 (s, 3H), 4.13 (t, *J* = 6.8 Hz, 1H), 6.50 (br. s, 1H), and 7.71–8.01 (m, 40H); ^11^B {^1^H} NMR (DMSO-d_6_, δ, ppm): −31.4, −27.4, −21.5, −3.8, and −0.1; ^13^C NMR (DMSO-d_6_, δ, ppm): 14.2, 24.2, 28.3, 29.6, 33.4, 51.0, 52.7, 68.1, 69.6, 71.9, 130.3, 130.5, 134.4, 134.6, 135.3, 169.7, and 171.7; and IR (KBr, cm^−1^): 2445 (ν(B-H)) and 1746 ((ν(C=O)).

(h) Compound (Ph_4_P)_2_**4** was prepared according to a procedure similar to that used to prepare compound (Ph_4_P)_2_**1** using 0.25 g (0.27 mmol) of (Ph_4_P)_2_[B_10_H_9_O(CH_2_)_2_O(CH_2_)_2_CH_2_COOH] and 0.06 g (0.27 mmol) of another aminoacid HCl · H-Pld-OCH_3_.

The following results were obtained: ^1^H NMR (DMSO-d_6_, δ, ppm): −0.99–0.75 (m, 9H), 0.80–1.21 (m, 2H), 1.59–1.73 (m, 1H), 1.83–2.13 (m, 3H), 2.28–2.32 (m, 1H), 2.56–2.64 (m, 1H), 3.16–3.48 (m, 10H), 3.76 (s, 3H), 3.99–4.19 (m, 2H), 7.70–8.00 (m, 40H), and 8.75 (br.s, 1H); ^11^B {^1^H} NMR (DMSO-d_6_, δ, ppm): −31.7, −27.0, −21.3, −5.2, −3.3, and −0.4; ^13^C NMR (DMSO-d_6_, δ, ppm): 27.6, 29.6, 31.7, 34.4, 38.2, 51.1, 52.7, 60.2, 70.1, 70.7, 72.0, 130.3, 130.5, 134.4, 134.6, 135.3, 169.7, 175.6, and 178.1; and IR (KBr, cm^−1^): 2447 (ν(B-H)) and 1749 ((ν(C=O)).

(i) Synthesis of sodium methyl 2-[4-(2-methoxyethoxy)butanamido]-3-(2-oxopyrrolidin-3-yl)propanoate-nonahydro-closo-decaborate Na_2_[B_10_H_9_O(CH_2_)_2_O(CH_2_)_2_CH_2_COPld] Na_2_**4**. (Ph_4_P)_2_**4** (0.222 g, 0.20 mmol) was dissolved in 2 mL of methanol. A solution of Na[BPh_4_] (0.137 g, 0.40 mmol) in 2 mL of methanol was added to this solution, and a white precipitate formed. The precipitate was filtered, and the solution was evaporated to obtain a white hygroscopic powder, which was dried in high vacuum (yield, 0.047 g (49%)).

The following results were obtained: ^1^H NMR (DMSO-d_6_, δ, ppm): −0.99–0.75 (m, 9H), 0.80–1.21 (m, 2H), 1.59–1.73 (m, 1H), 1.83–2.13 (m, 3H), 2.28–2.32 (m, 1H), 2.56–2.64 (m, 1H), 3.16–3.48 (m, 10H), 3.76 (s, 3H), 3.99–4.19 (m, 2H), and 8.81 (br.s, 1H); ^13^C NMR (DMSO-d_6_, δ, ppm): 27.6, 29.6, 31.7, 34.4, 38.2, 51.1, 52.7, 60.2, 70.1, 70.7, 72.0, 169.7, 175.6, 178.1; and IR (KBr, cm^−1^): 2456 (ν(B-H)) and 1714 ((ν(C=O)).

### Molecular Docking

Molecular docking was performed using the Neurosnap online service, which allows any user with a Neurosnap account to run and access DiffDock-L (https://neurosnap.ai/service/DiffDock-L) (assessed on 15 October 2024). DiffDock-L is a new and improved version of DiffDock, a state-of-the-art molecular docking and drug binding structure prediction method. This service uses DiffDock-L and AutoDock VINA to predict protein–ligand interactions with high accuracy [37]. The structure of the transmembrane region of the M2 channel (PDB code: 2KIH) was obtained from the RCSB PDB open database (https://www.rcsb.org/) (assessed on 15 October 2024). The ligand structure (**1**^2−^ or **2**^2−^) was added to DiffDock-L as a SMILES string as suggested by the developers. The SMILES string of **1**^2−^ or **2**^2−^ was generated in the SMILES generator service (https://www.cheminfo.org/flavor/malaria/Utilities/SMILES_generator___checker/index.html) (assessed on 15 October 2024).

## 4. Conclusions

New *closo*-decaborate derivatives [B_10_H_9_–O(CH_2_)_2_O(CH_2_)_3_C(O)–L–OCH_3_] (L = Trp (**1**), His (**2**), Met (**3**), Pld (**4**)) were synthesized and isolated as sodium salts. These anions feature a pendant functional group -L-OCH_3_, which is bonded to the boron cluster anion via an alkoxy spacer. Toxicity and antiviral activity against influenza A virus of the compounds were evaluated. All the compounds were found to be low in toxicity to the MDCK cell monolayer. Compound Na_2_**1** exhibited a high degree of inhibition against the influenza virus strain A(H1N1)pdm09, with the 50% inhibitory dose of 5.0 μg/mL, and thus the chemotherapeutic index (Si) was 16. For compound Na_2_**2**, Si was lower and was 8. The in silico studies preliminarily classify these compounds as blockers of the ion-selective channel M2, due to their structure, in which the boron cluster cage is a membranotropic carrier for the functional group of the amino acid. The proposed model compounds are inhibitors representing a new class of blockers of ion channels of viruses (viroporins). The proposed compounds, due to their low toxicity and high solubility in aqueous solutions, can be a starting point for the creation of new inhibitors of viroporins of RNA-containing viruses on their basis. In the future, they can be used as drugs in the complex therapy of infections caused by RNA-containing viruses.

## Data Availability

Data are provided within the manuscript or Supplementary Information files.

## Supplementary Materials

The following supporting information can be downloaded at: https://www.mdpi.com/article/10.3390/molecules29245886/s1, Figure S1: Synthesis of oseltamivir derivative with closo-dodecaborane - 1,12-dicarba-closo-dodecaborane(12)-1-prop-3-yl (3R,4R,5S)-4-acetamido-5-azido-3-(1-ethylpropoxy )-cyclohex-1-ene-1-carboxylate; Figure S2: Amino acid derivative of the closo-decaborate anion [B10H9–O(CH2)4C(O)–His–OMe]2–; Figure S3: Complex of the crystallographic structure of the M2 channel of the influenza A virus (2KIH) and the synthetic blocker of ion channel function Na_2_**1**, as well as details of biological experiments and ^1^H, ^11^B, and ^13^C NMR, IR spectroscopy data.

## Abbreviations

Trp	tryptophan
His	histidine
Pld	alanine-2-oxopyrrolidin-3-yl
Met	methionine
MDCK	Madin-Darby canine kidney
NMR	Nuclear magnetic resonance
CT_50_	50% cytotoxic concentration
OD	optical density
NMM	N-methylmorpholine
IBCF	iso-butyl chloroformate
