# Supplementary material for: In Vitro Study of Antiviral Properties of Compounds Based on 1,4-Dioxane Derivative of Closo-Decaborate Anion with Amino Acid Ester Residues Against Influenza Virus A/IIV-Orenburg/83/2012(H1N1)pdm09
Source: Molecules. 2024 Dec 13;29(24):5886. doi: 10.3390/molecules29245886 (PMC11678584; doi:10.3390/molecules29245886)
Supplement: Supplementary file 1 [file molecules-29-05886-s001.zip › molecules-3334525-supplementary.pdf]

SUPPORTING INFORMATION

CONTENTS

Figure S1. Synthesis of oseltamivir derivative with closo-dodecaborane - 1,12-dicarba-closo-dodecaborane(12)-1-prop-3-yl (3R,4R,5S)-4-acetamido-5-azido-3-(1-ethylpropoxy)-cyclohex-1-ene-1-carboxylate. 2

Figure S2. (a) Amino acid derivative of the *closo*-decaborate anion $[B_{10}H_9–O(CH_2)_4C(O)–His–OMe]^{2–}$; (b) and (c) front and top views of the influenza A virus M2 proton channel molecular complex with the boron derivative are shown. Characteristic residues are highlighted in color: His37 (green) and Trp41 (blue). ...2

Figure S3. Complex of the crystallographic structure of the M2 channel of the influenza A virus (2KIH) and the synthetic blocker of ion channel function Na_2**1**. The docking results are shown from the side and from the top, as well as an enlarged fragment of the solution is present indicating the important amino acid residues in the pore of the M2 channel involved in the interaction with the inhibitor structure. 3

Antiviral activity 4

Determination of cytotoxic effect (MTT test) 4

Methods of characterization 5

NMR Spectroscopy data 5

IR spectroscopy data 26

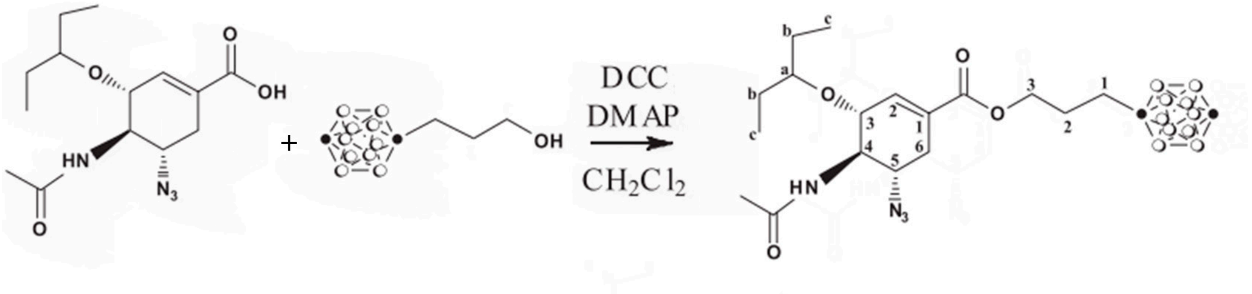

Figure S1. Synthesis of oseltamivir derivative with closo-dodecaborane - 1,12-dicarba-closo-dodecaborane(12)-1-prop-3-yl (3R,4R,5S)-4-acetamido-5-azido-3-(1-ethylpropoxy)-cyclohex-1-ene-1-carboxylate.

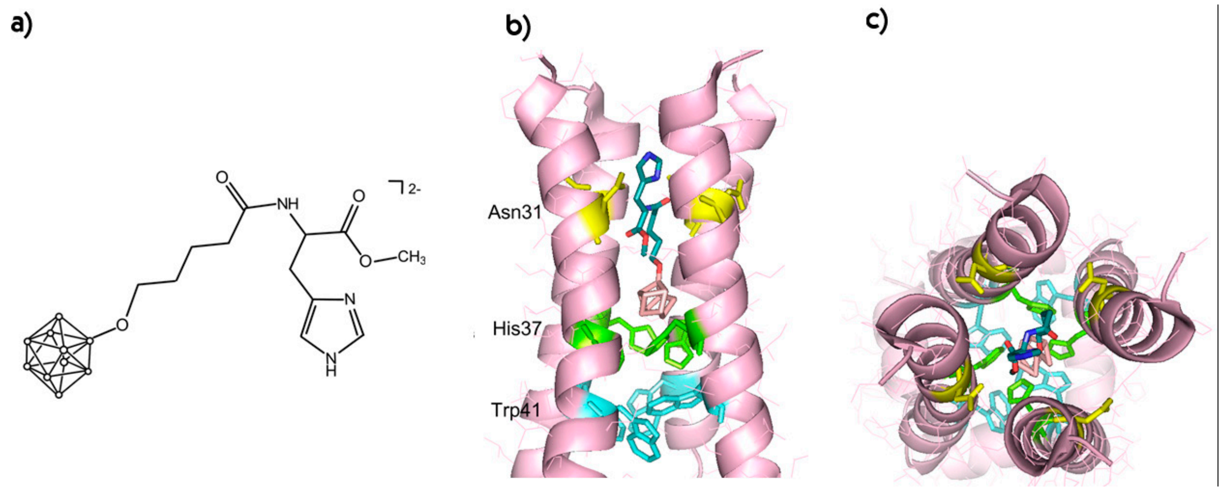

Figure S2. (a) Amino acid derivative of the *closo*-decaborate anion $[B_{10}H_9–O(CH_2)_4C(O)–His–OMe]^{2–}$; (b) and (c) front and top views of the influenza A virus M2 proton channel molecular complex with the boron derivative are shown. Characteristic residues are highlighted in color: His37 (green) and Trp41 (blue).

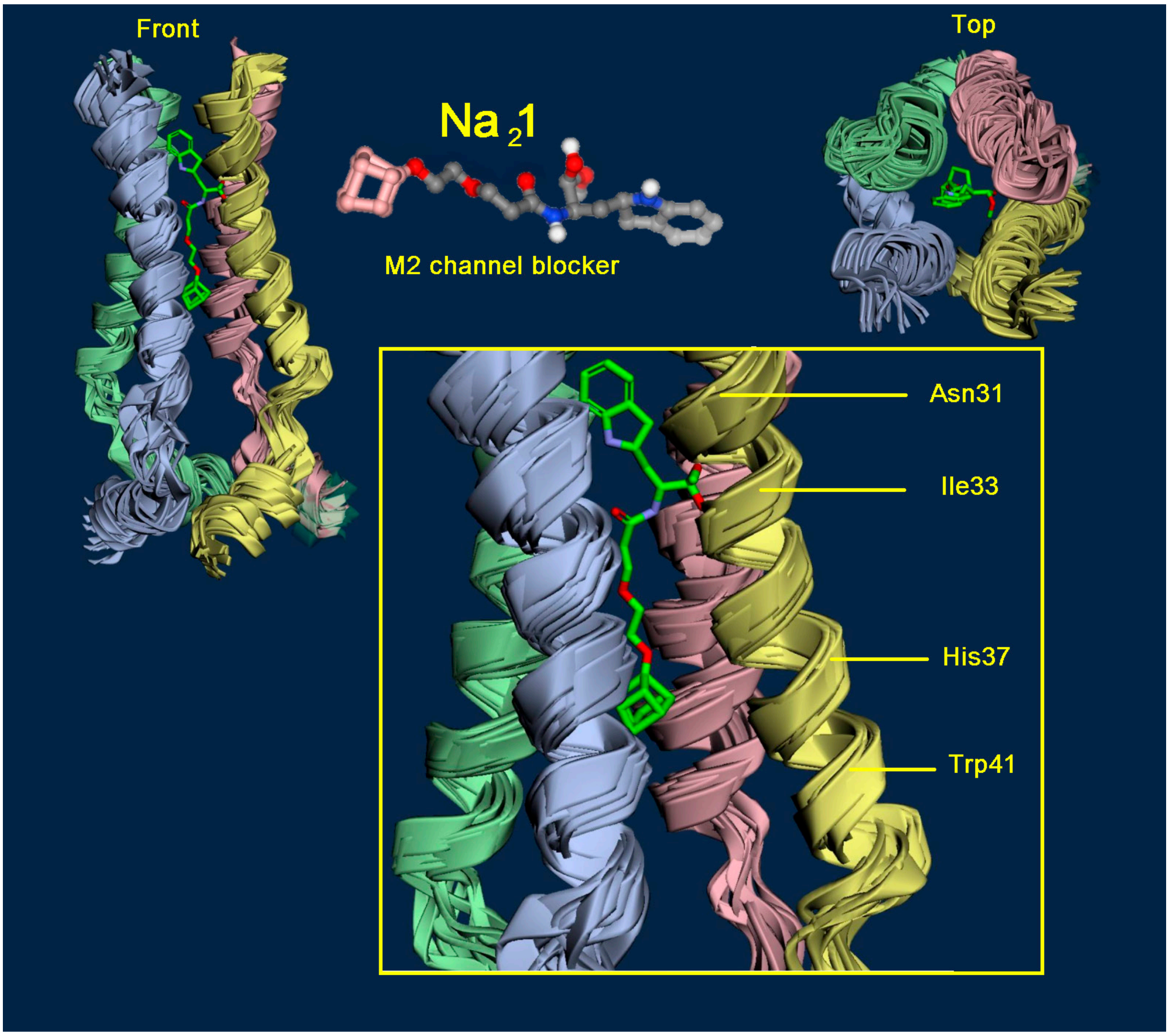

Figure S3. Complex of the crystallographic structure of the M2 channel of the influenza A virus (2KIH) and the synthetic blocker of ion channel function Na_2**1**. The docking results are shown from the side and from the top, as well as an enlarged fragment of the solution is present indicating the important amino acid residues in the pore of the M2 channel involved in the interaction with the inhibitor structure.

Antiviral activity

The antiviral activity of compounds $Na_2$1 and $Na_2$2 was evaluated using 96-well plates with a formed monolayer of MDCK tissue culture cells. Rimantadine was used as a control, and the synthetic compounds were added to the cell monolayer at concentrations of 5.0, 10.0, and 20.0 μg/mL simultaneously with the viral infection. The plates were incubated for 24 hours at 37°C, after which the reaction was halted by fixing the cells with 80% acetone in phosphate buffer. The ELISA assay was conducted following previously described methods [refs. indicated in the main text]. The percentage of viral inhibition was calculated using the following formula:

$$100 - \left(\frac{OD_{exp} - OD_{cell\ contr}}{OD_{viral\ contr} - OD_{cell\ contr}}\right) \times 100\%, \quad (1)$$

where OD_{exp} is the optical density of the experimental well (with the compound) at 492 nm, $OD_{\mathrm{cell\ contr}}$ is the optical density of the cell control, $OD_{viral\ contr}$ is the optical density of the virus control.

Determination of cytotoxic effect (MTT test)

The cytotoxicity of compounds $Na_2$1 and $Na_2$2 was assessed by adding different concentrations of the compounds to a monolayer of MDCK tissue culture cells in 96-well plates and incubating at 37°C. Initially, the cell monolayer was examined under a microscope. The concentration that caused degeneration of 50% of the cells compared to the control was recorded as the average toxic concentration (CT_{50}).

Additionally, cytotoxicity was evaluated using a colorimetric assay to measure cell metabolic activity with the dye 3-(4,5-dimethylthiazol-2-yl)-2,5-diphenyl-tetrazolium bromide (MTT). The test compound was added to the cells at concentrations of 40.0, 80.0, 160.0, 320.0, and 640.0 μg/mL (8 wells per concentration) in Eagle's MEM nutrient medium with L-glutamine and a double set of amino acids. The plates were incubated in a CO_2 incubator at 37°C for 48 h, with daily visual inspections under an optical microscope.

Following incubation, the medium was removed, and 20.0 μL of MTT solution (5.0 mg/mL, 100 μg of MTT) was added to each well. The plates were further incubated at 37°C for 2-4 h, during which dark blue formazan crystals formed in the cells. After incubation, the medium was removed using a multichannel pipette, revealing a blue coating of formazan crystals on the bottom of the wells. To dissolve the formazan crystals, 150.0 μL of dimethyl sulfoxide (DMSO) was added to each well, and the plate was shaken for 5 min.

The optical density (OD) of the solutions in each well was recorded using an automatic spectrophotometer at a wavelength of 490 nm. The cell viability was assessed by comparing the OD of the experimental wells with different drug concentrations with the OD of the control wells (cells without the drug).

Methods of characterization

IR spectra of the compounds were recorded on an INFRALUM FT-02 Fourier transform IR spectrometer in the range 400–4000 cm^{-1}. Samples were prepared in the form of tablets from a mixture of the test compound and potassium bromide (KBr).

^{1}H, ^{11}B, ^{13}C NMR spectra of solutions of compounds in DMSO-d_6 were recorded on a Bruker DPX-300 NMR spectrometer (Germany) at frequencies of 300.3, 96.32 and 75.49 MHz, respectively, with internal stabilization for deuterium.

NMR Spectroscopy data

NMR spectra were measured a 303K on a Bruker DPX 300 spectrometer operating at 300.13 MHz for proton nuclei, 75.5 MHz for carbon nuclei and 96.3 MHz for boron nuclei. Proton chemical shifts are expressed in parts per million (ppm, δ scale) and are referenced to residual protium in the NMR solvent (DMSO-d_6: 2.50). Carbon chemical shifts are expressed in parts per million (ppm, δ scale) and are referenced to carbon in the NMR solvent (DMSO-d_6: 39.52). ^{1}H NMR spectroscopic data are reported as follows: chemical shift in ppm (multiplicity, spin-spin coupling constants J (Hz), integration intensity). The multiplicities are abbreviated with s (singlet), d (doublet), t (triplet), broad (br.), combinations thereof, and m (multiplet). In case of combined multiplicities, the multiplicity with the larger coupling constant is stated first. Except for complex and overlapping multiplets, where a resonance range is given, the chemical shift of all other symmetric signals is reported as the center of the resonance multiplet. ^{13}C NMR spectroscopic data are reported as follows: chemical shift in ppm. ^{11}B NMR spectroscopic data are reported as follows: chemical shift in ppm. Spectra processing were performed using Bruker TopSpin 2.1 and MestReNova Version 14.3.1-31739 software packages.

Copies of ^{1}H, $^{13}C\{^{1}H\}$ and $^{11}B\{^{1}H\}$

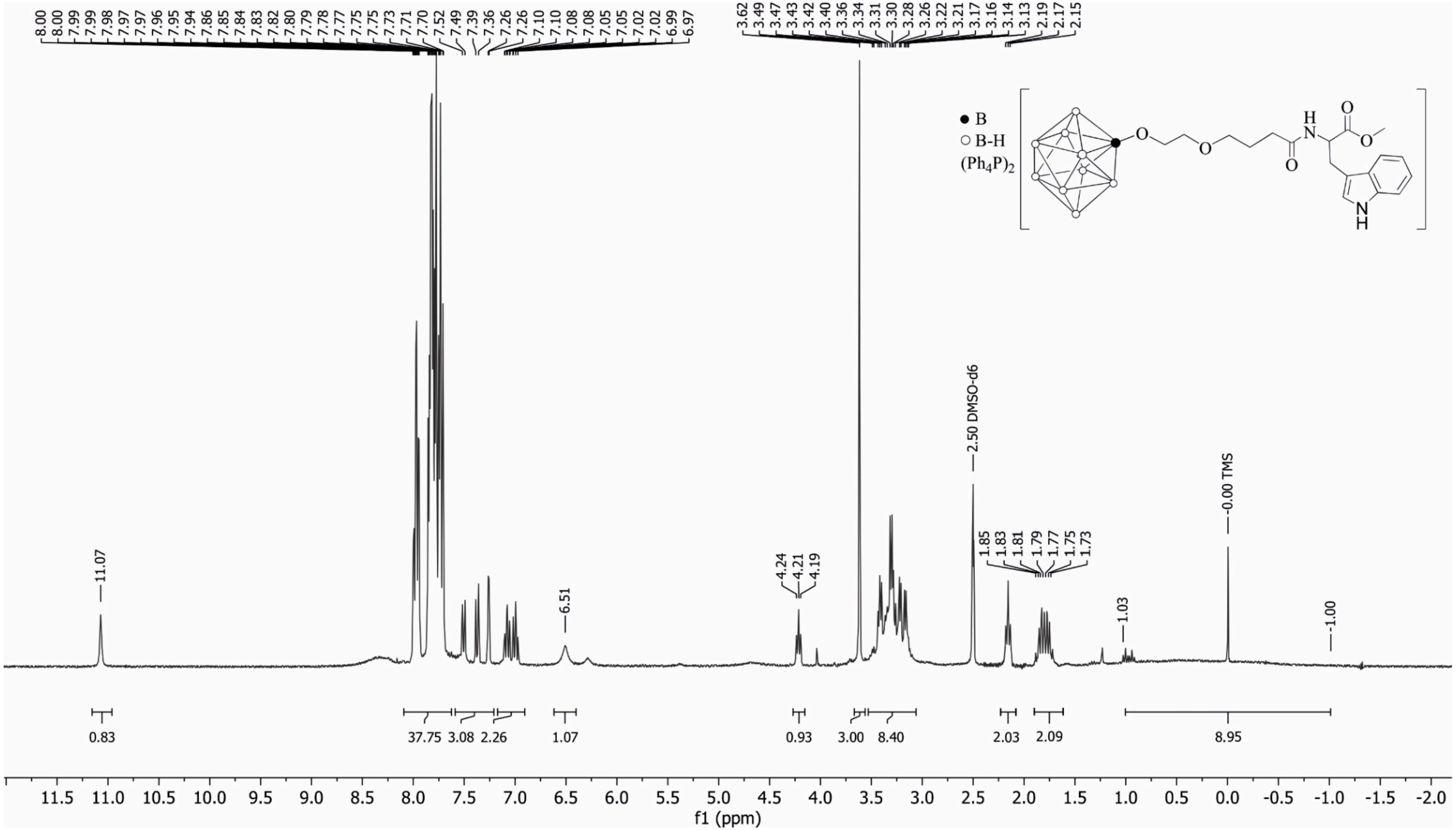

^{1}H NMR of **$(Ph_4P)_2$1**

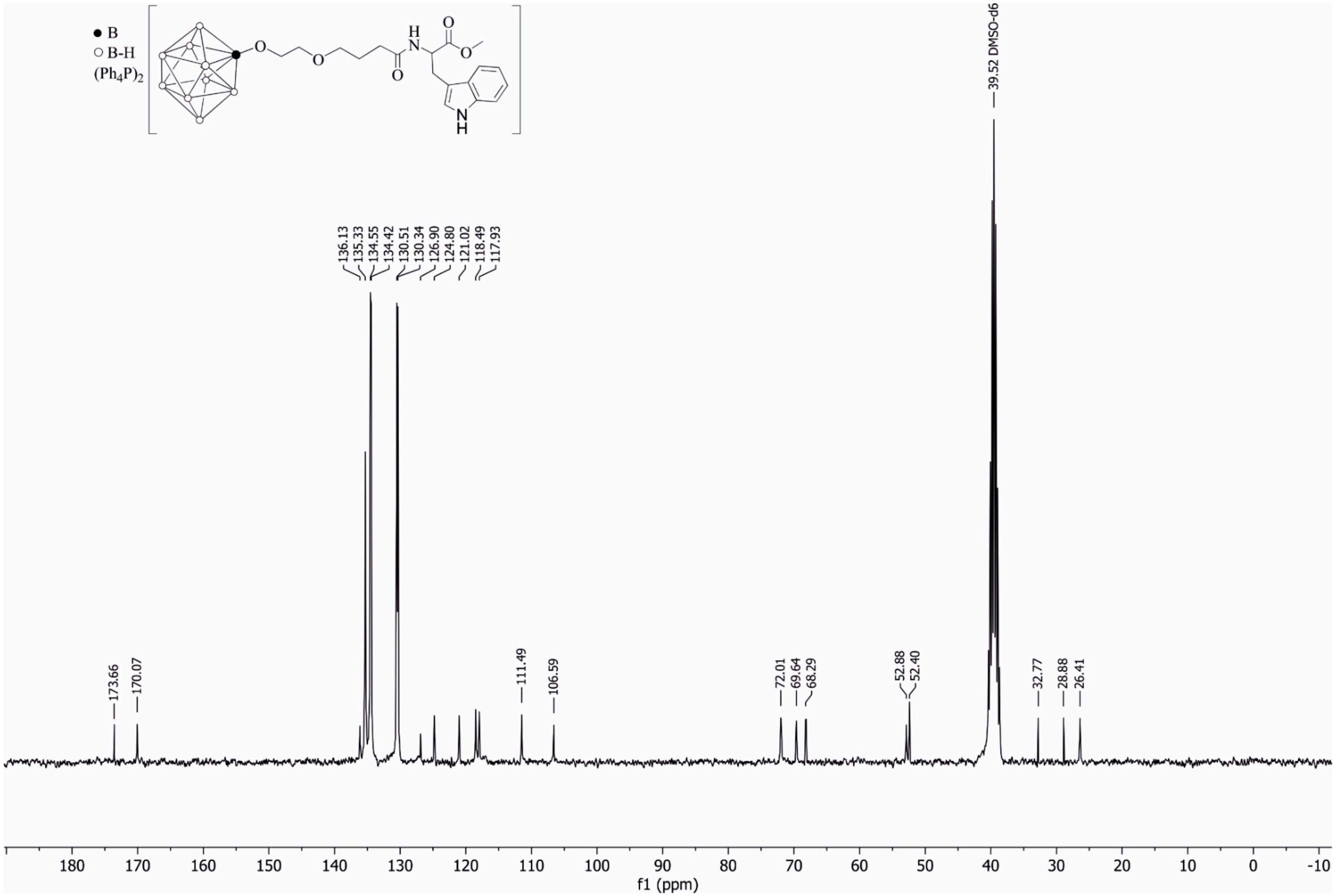

^{13}C{^{1}H} NMR of **$(Ph_4P)_2$1**

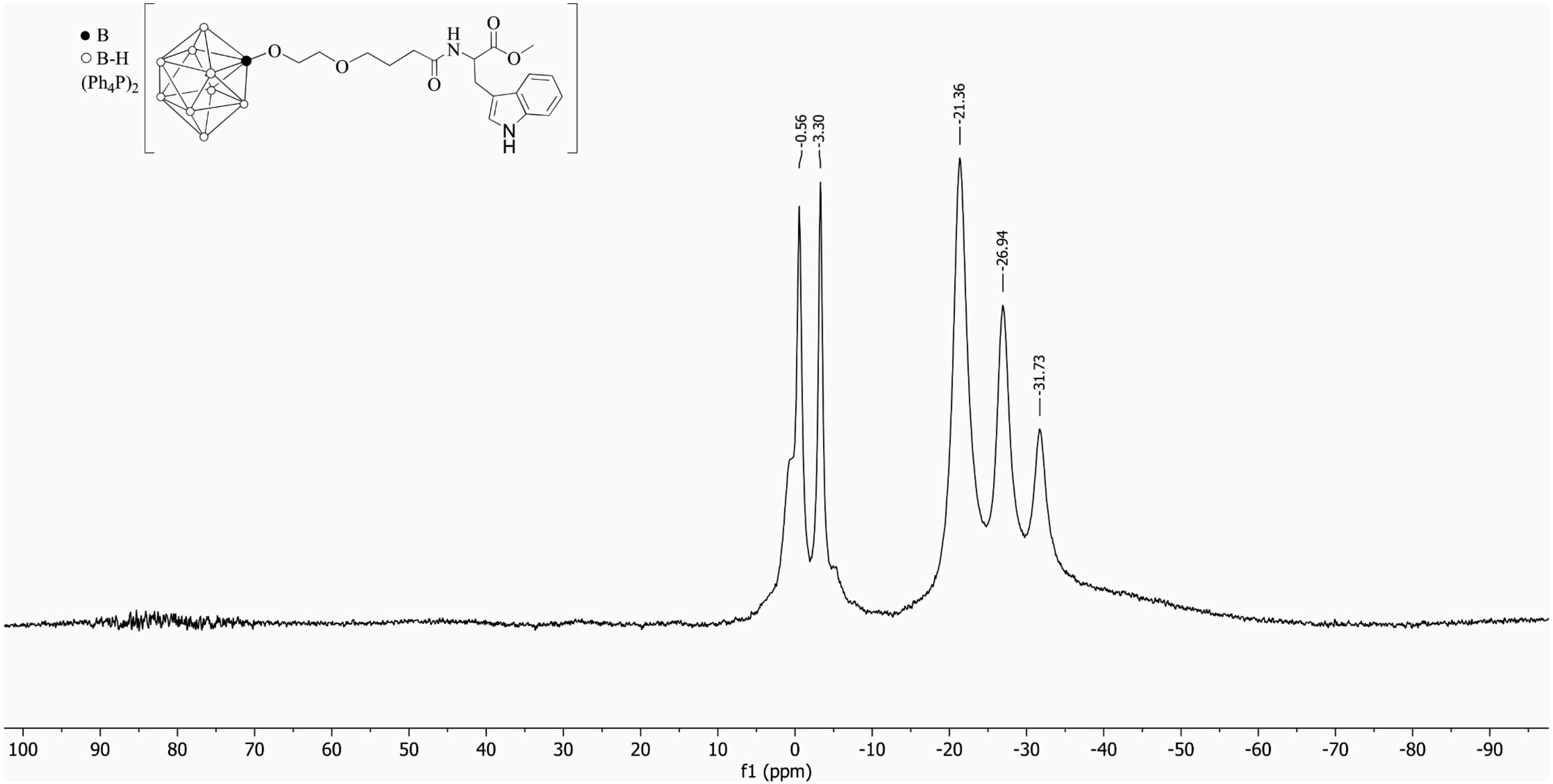

$^{11}B\{^1H\}$ NMR of **$(Ph_4P)_2$1**

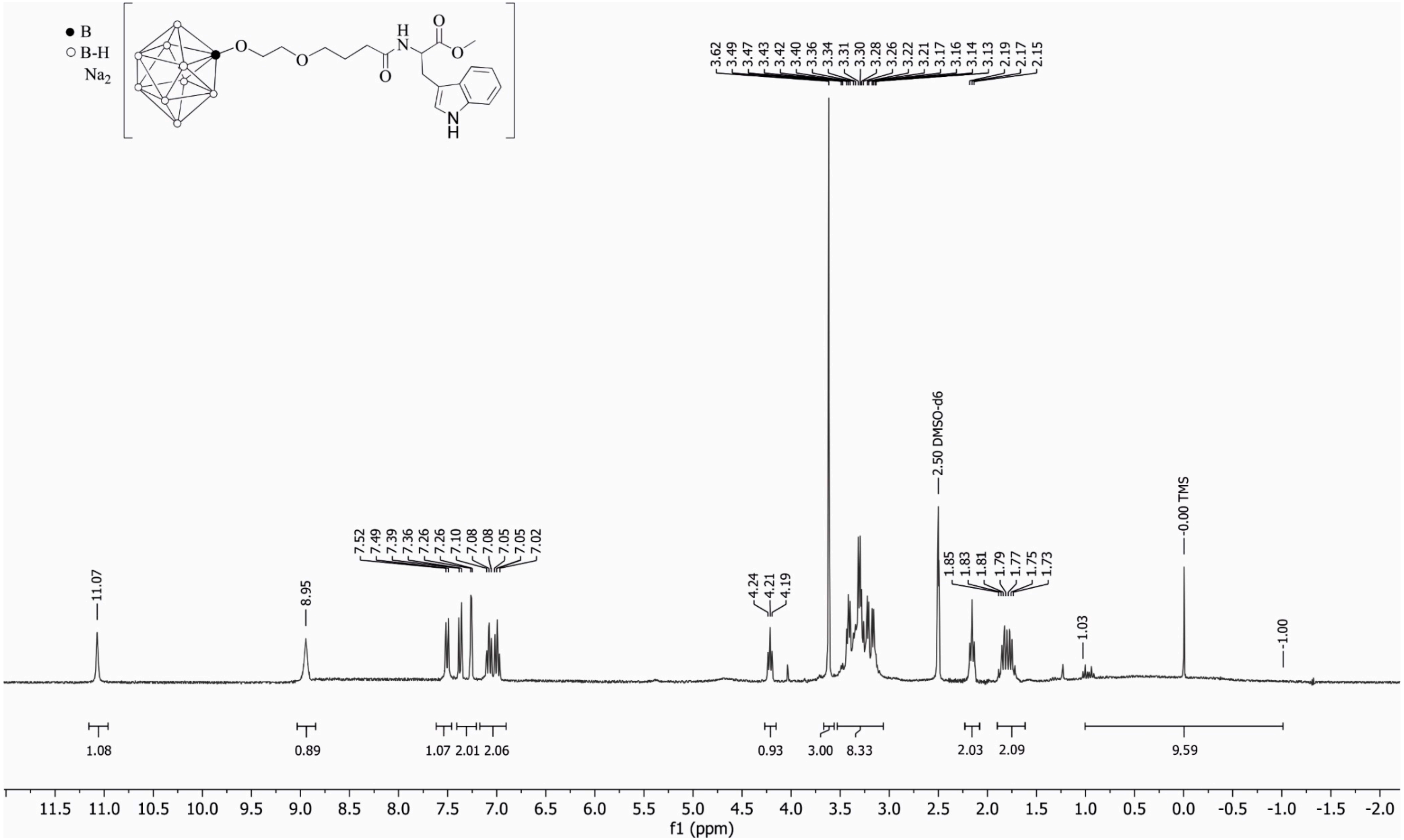

^{1}H NMR of **Na$_2$1**

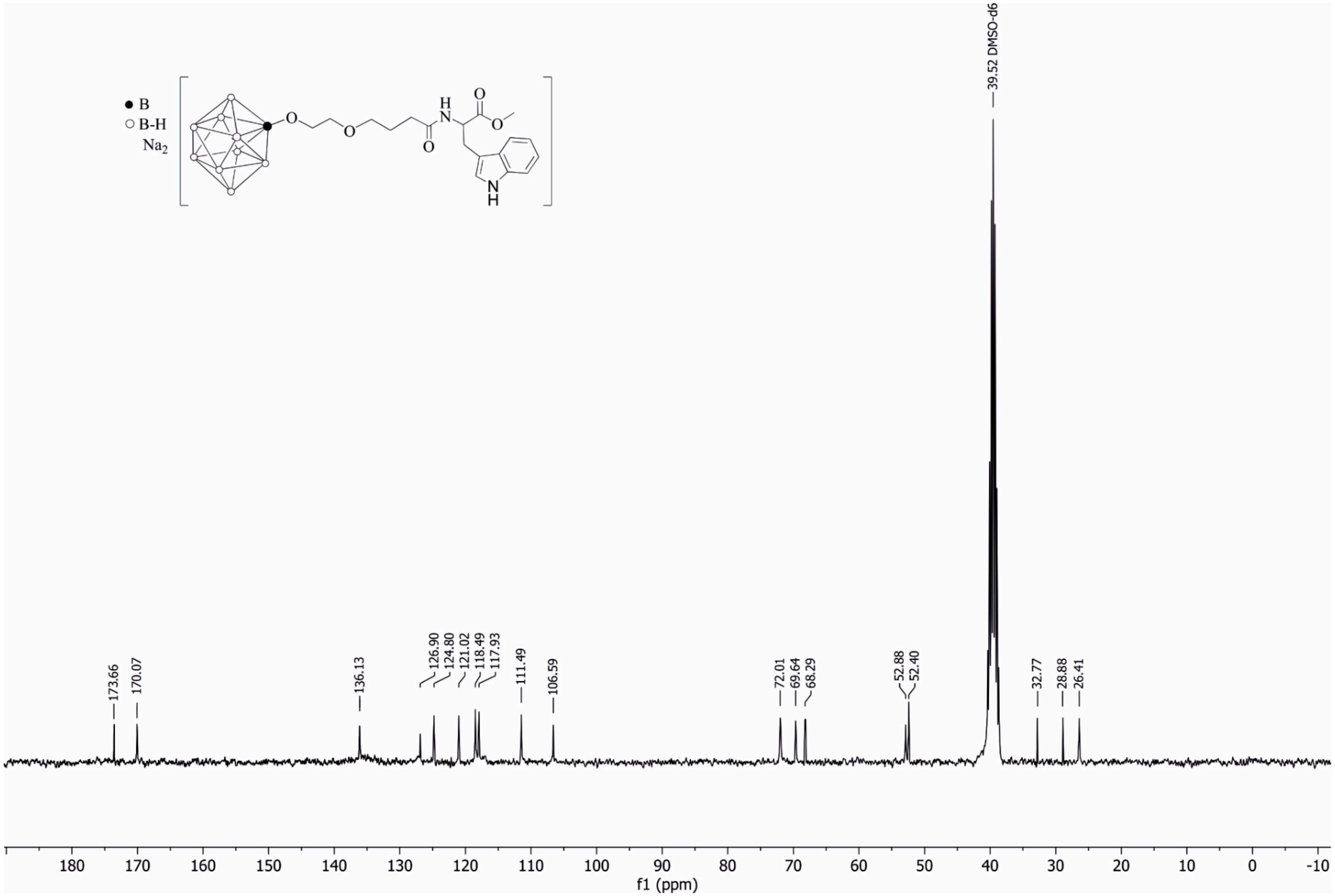

$^{13}C\{^{1}H\}$ NMR of **Na₂1**

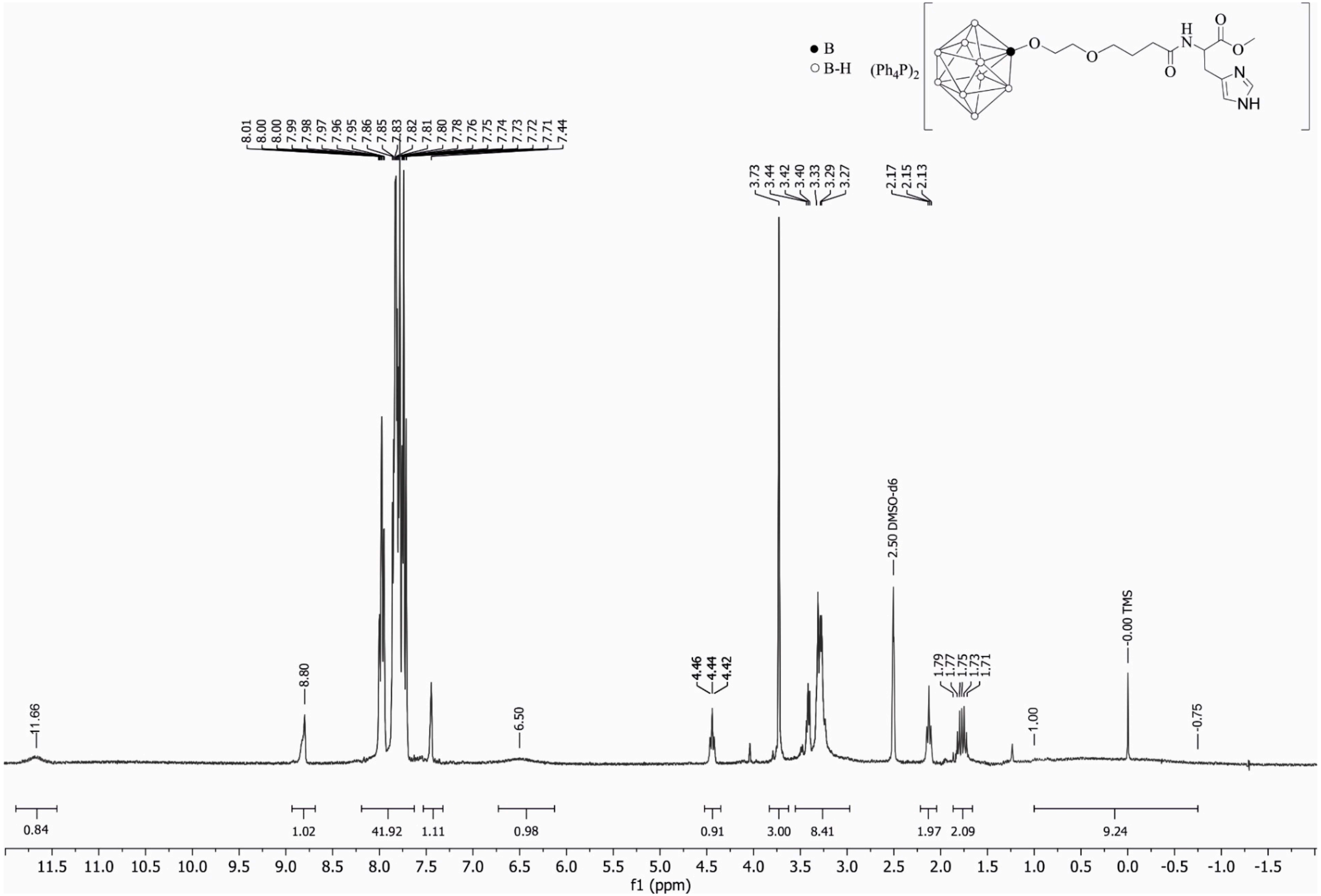

^{1}H NMR of **(Ph$_4$P)$_2$2**

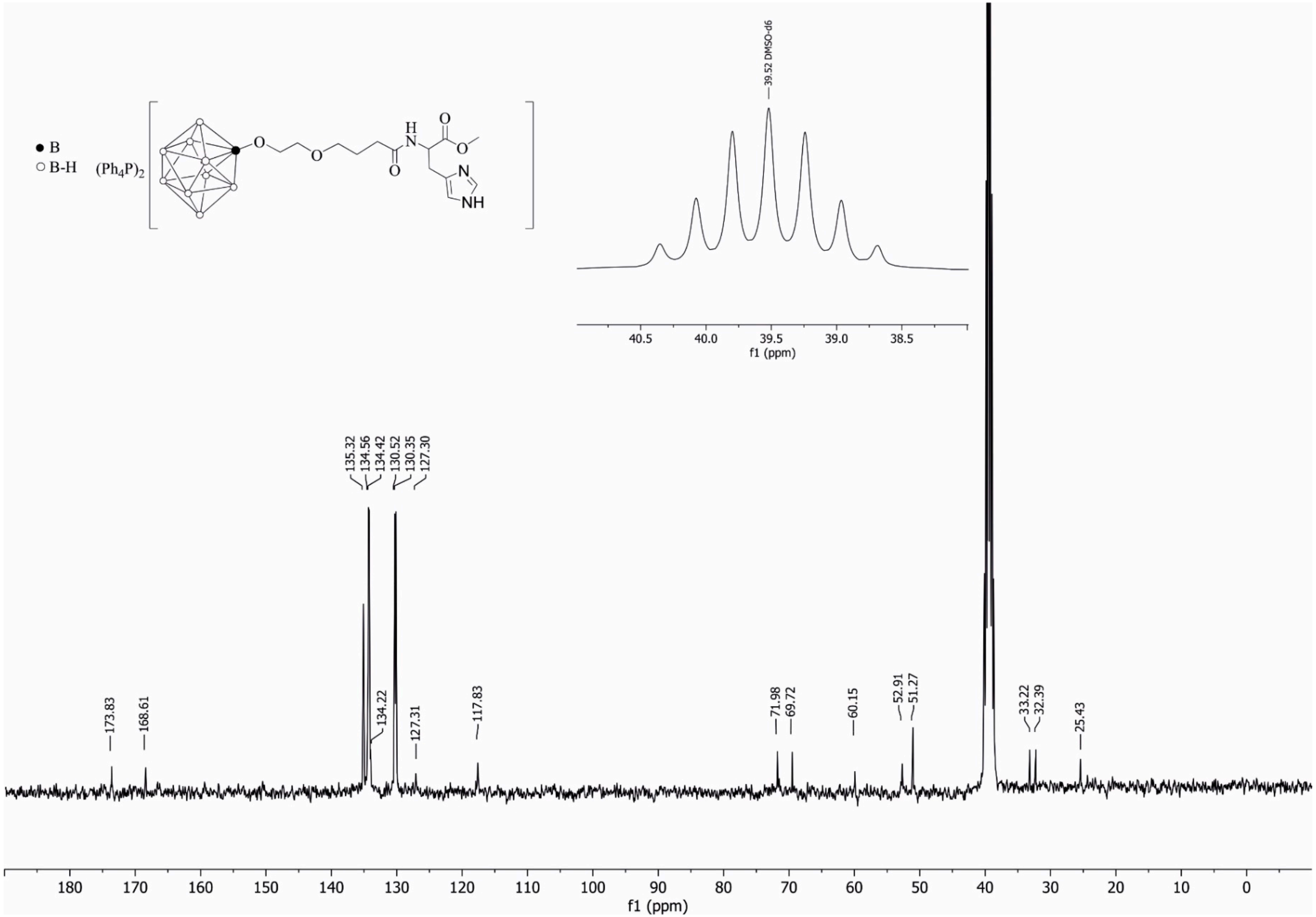

$^{13}C\{^1H\}$ NMR of **$(Ph_4P)_2$2**

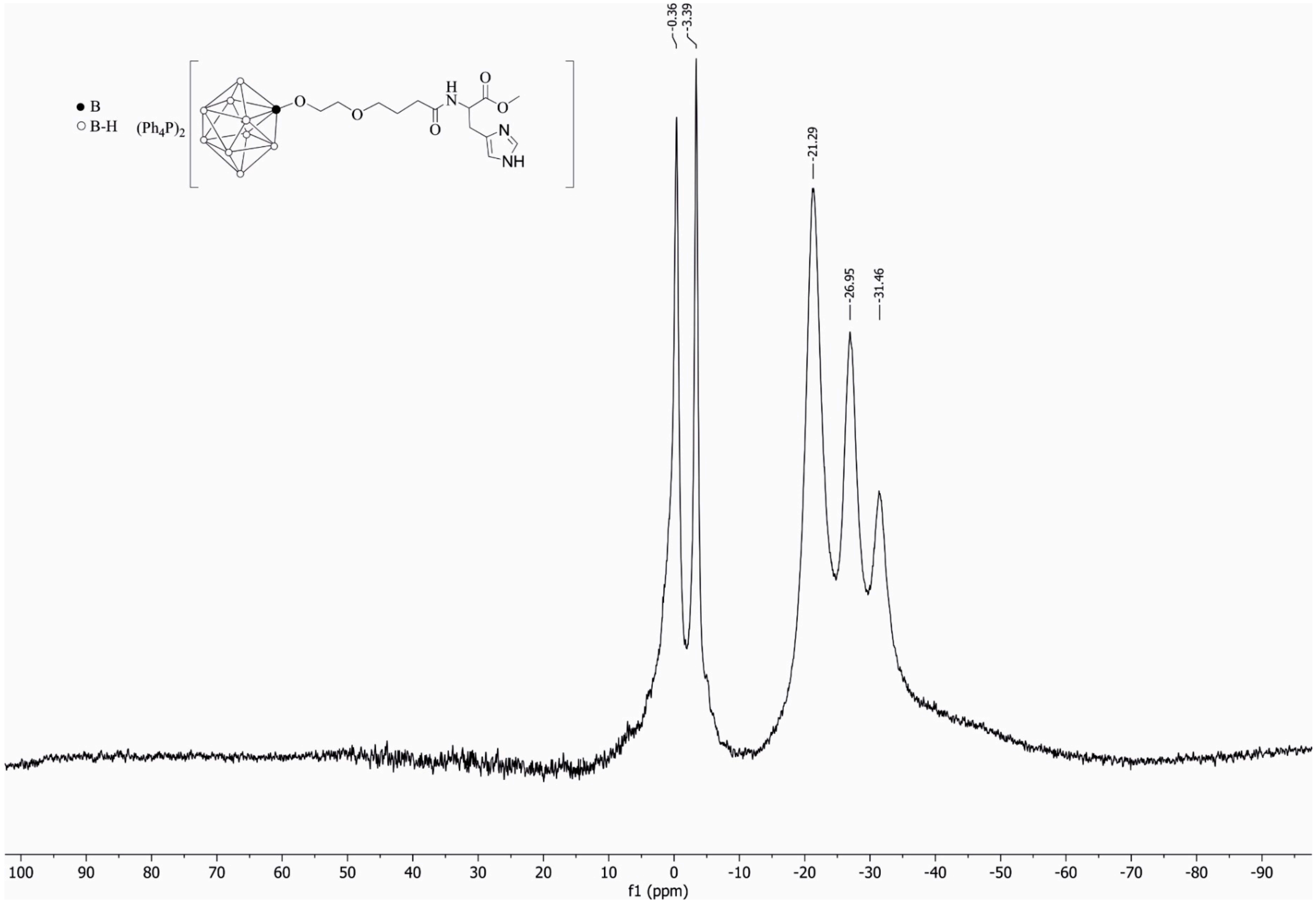

$^{11}B\{^{1}H\}$ NMR of **$(Ph_4P)_2$2**

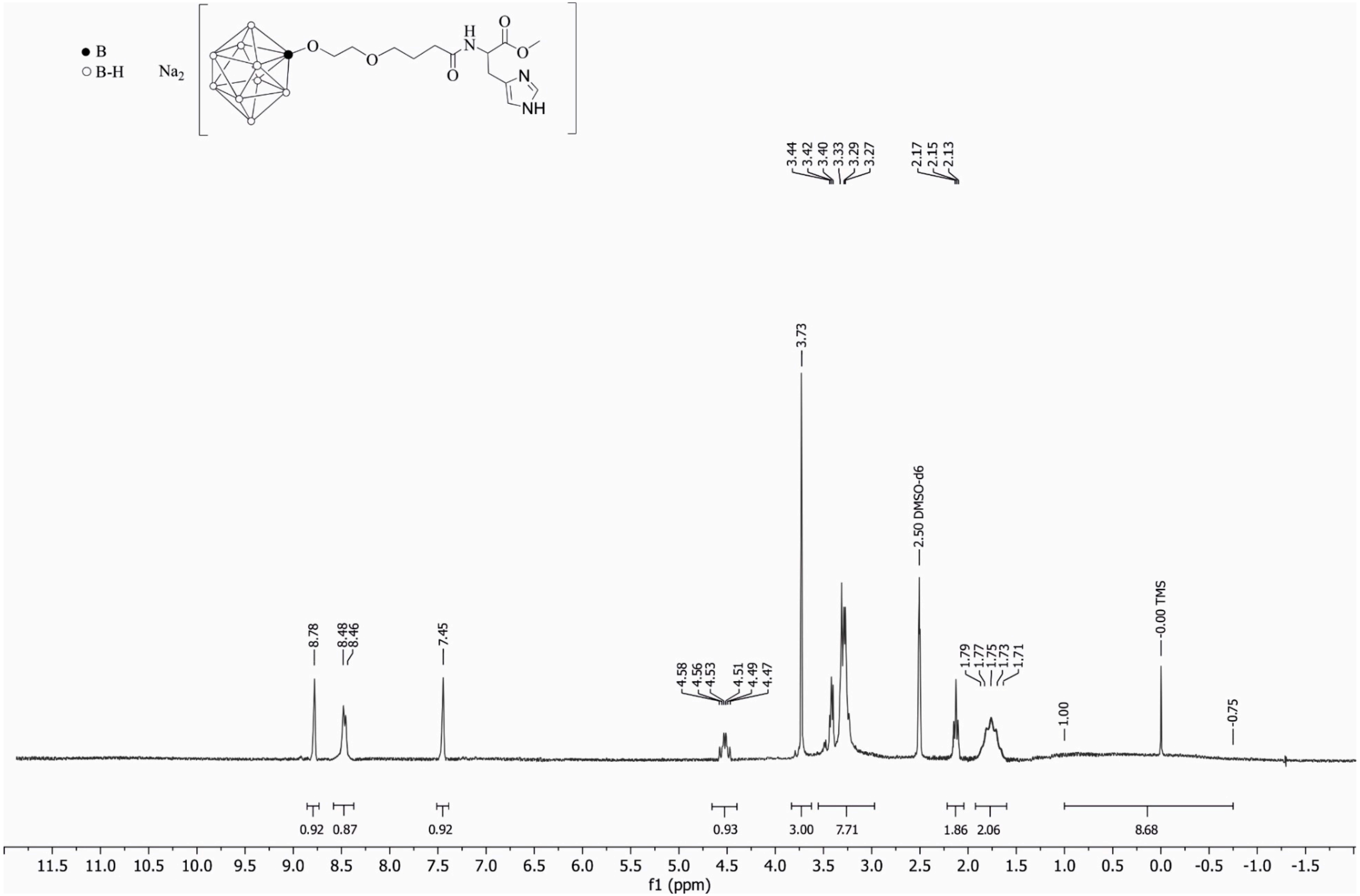

^{1}H NMR of **Na$_2$2**

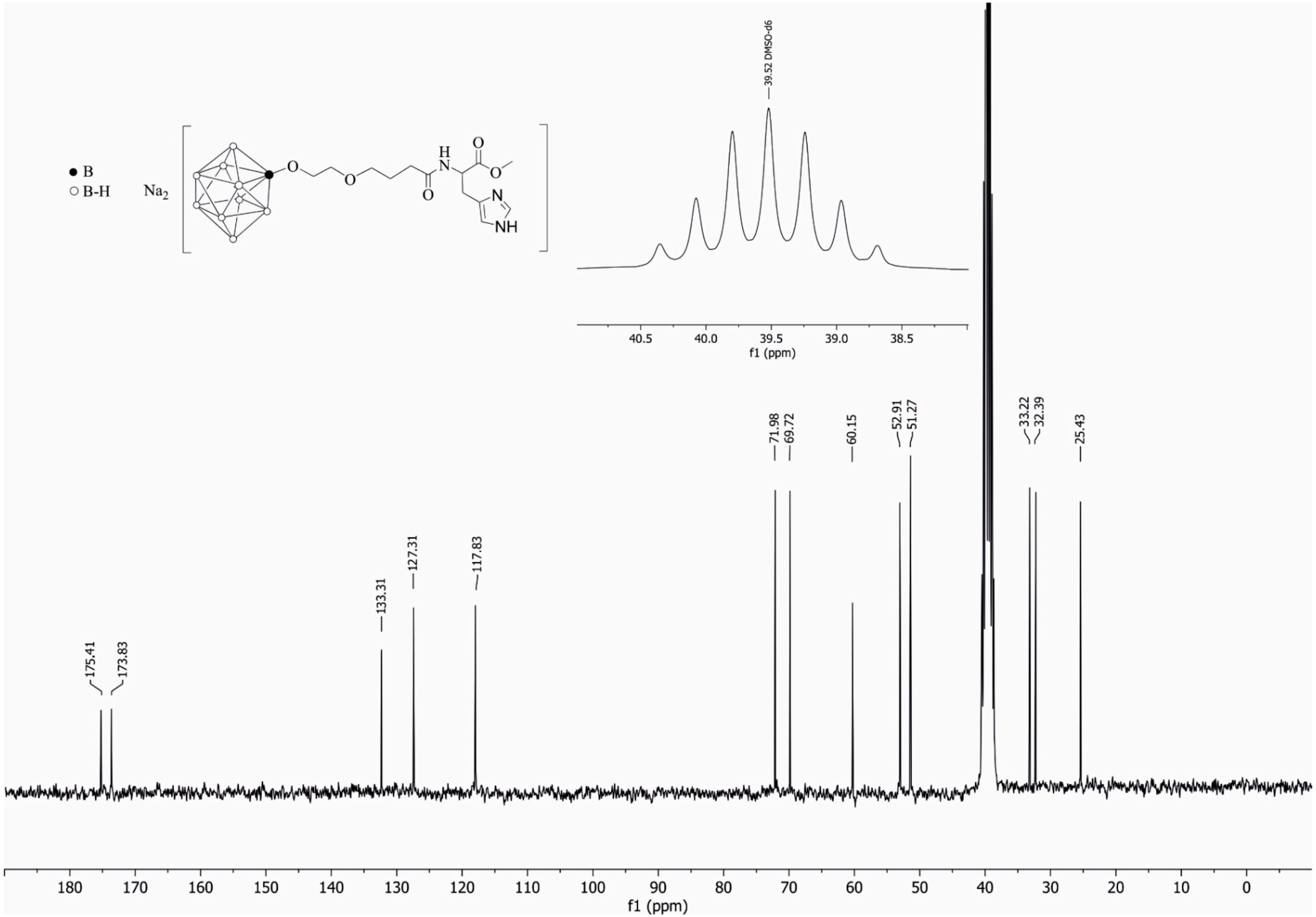

$^{13}C\{^{1}H\}$ NMR of **$Na_2$2**

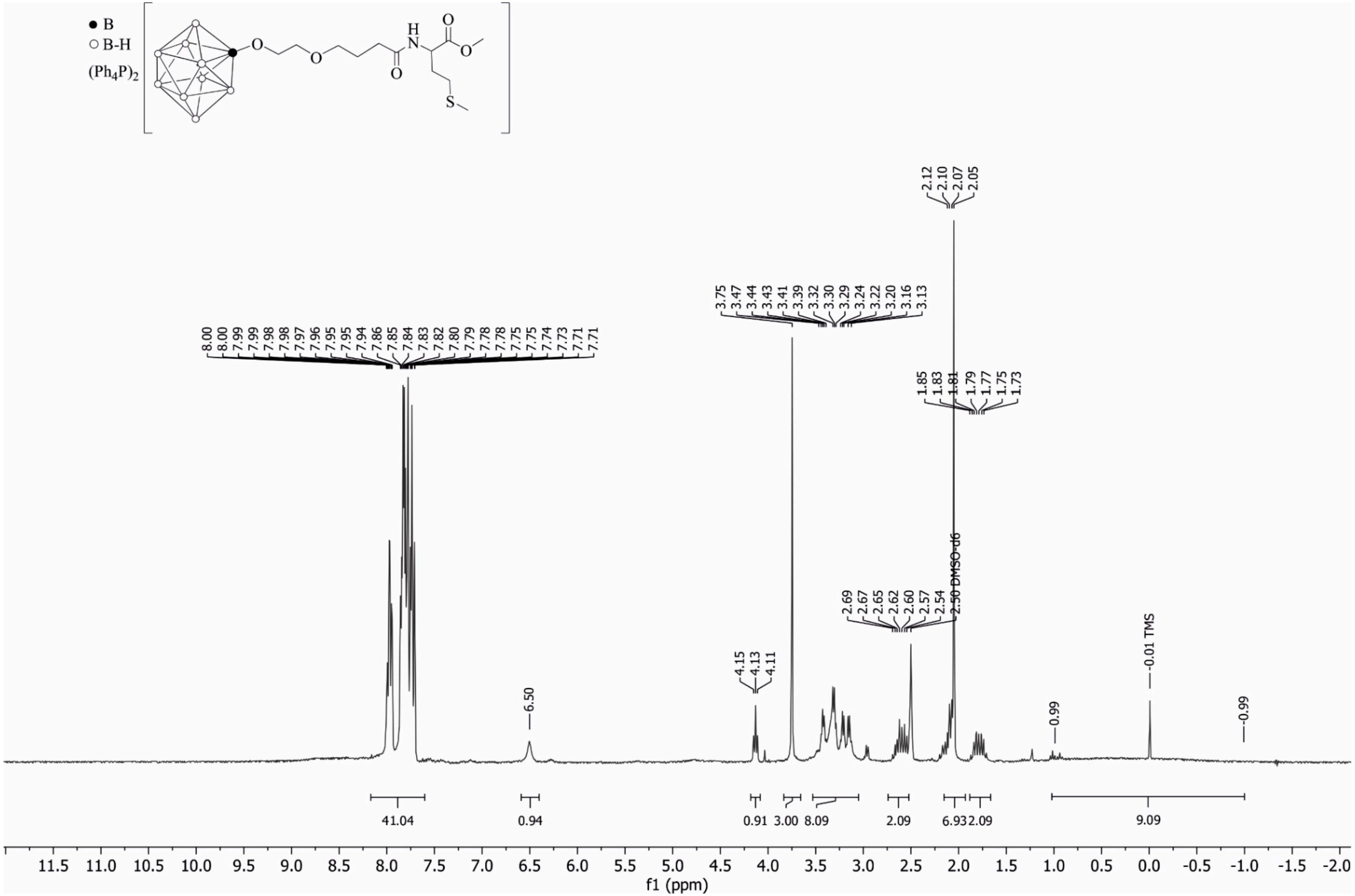

^{1}H NMR of **$(Ph_4P)_2$3**

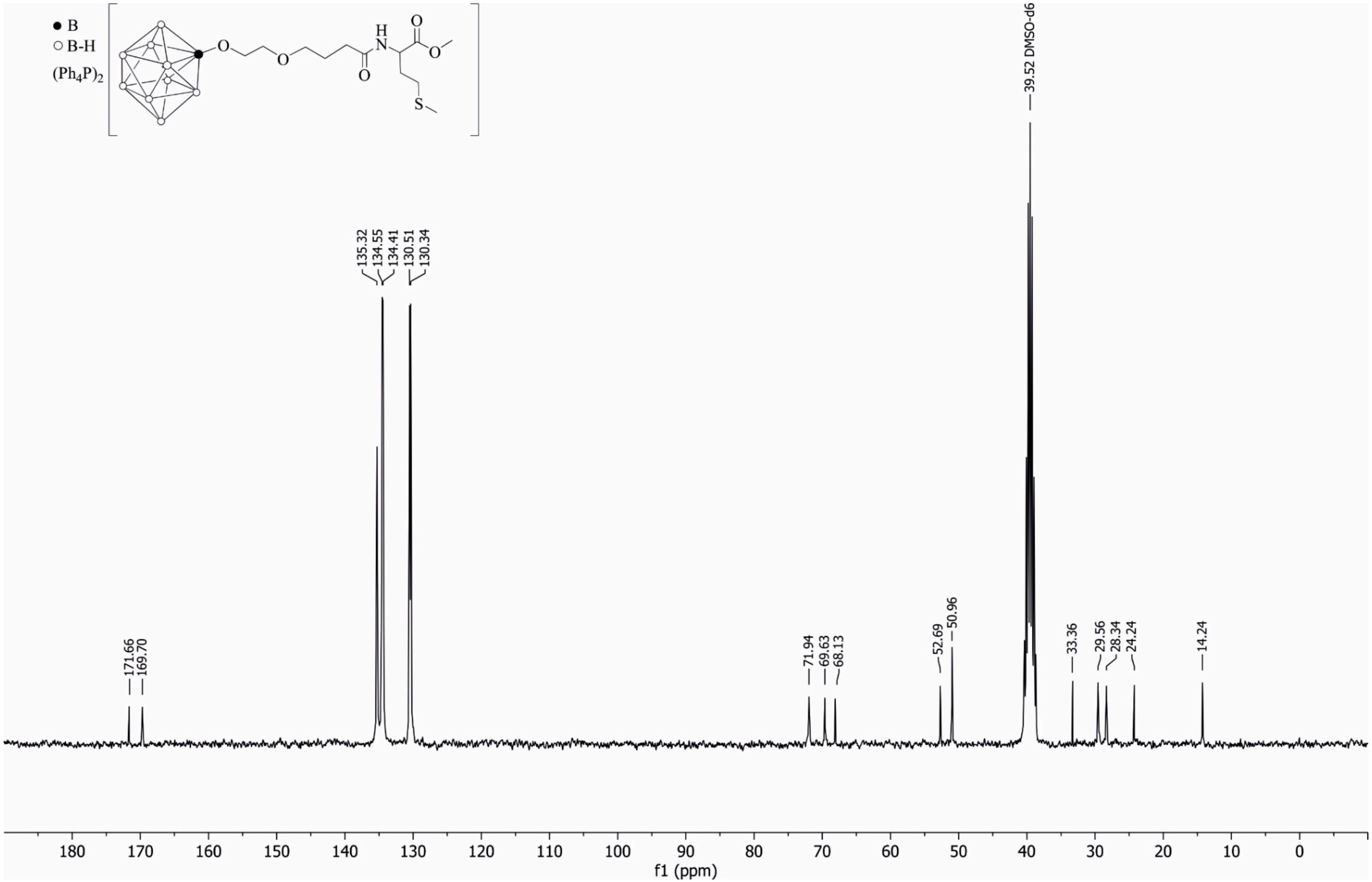

$^{13}C\{^{1}H\}$ NMR of **$(Ph_4P)_2$3**

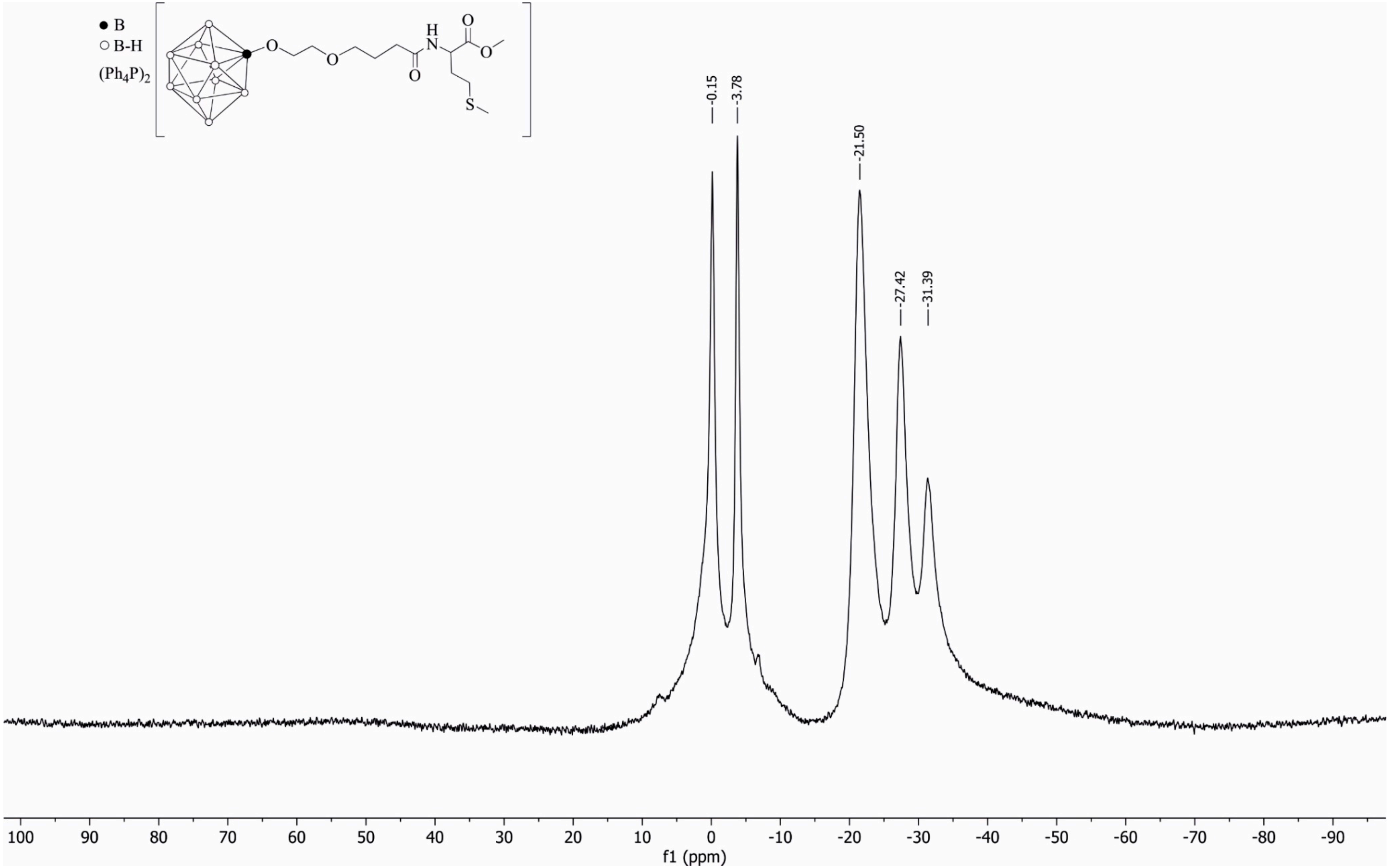

$^{11}B\{^{1}H\}$ NMR of **$(Ph_4P)_2$3**

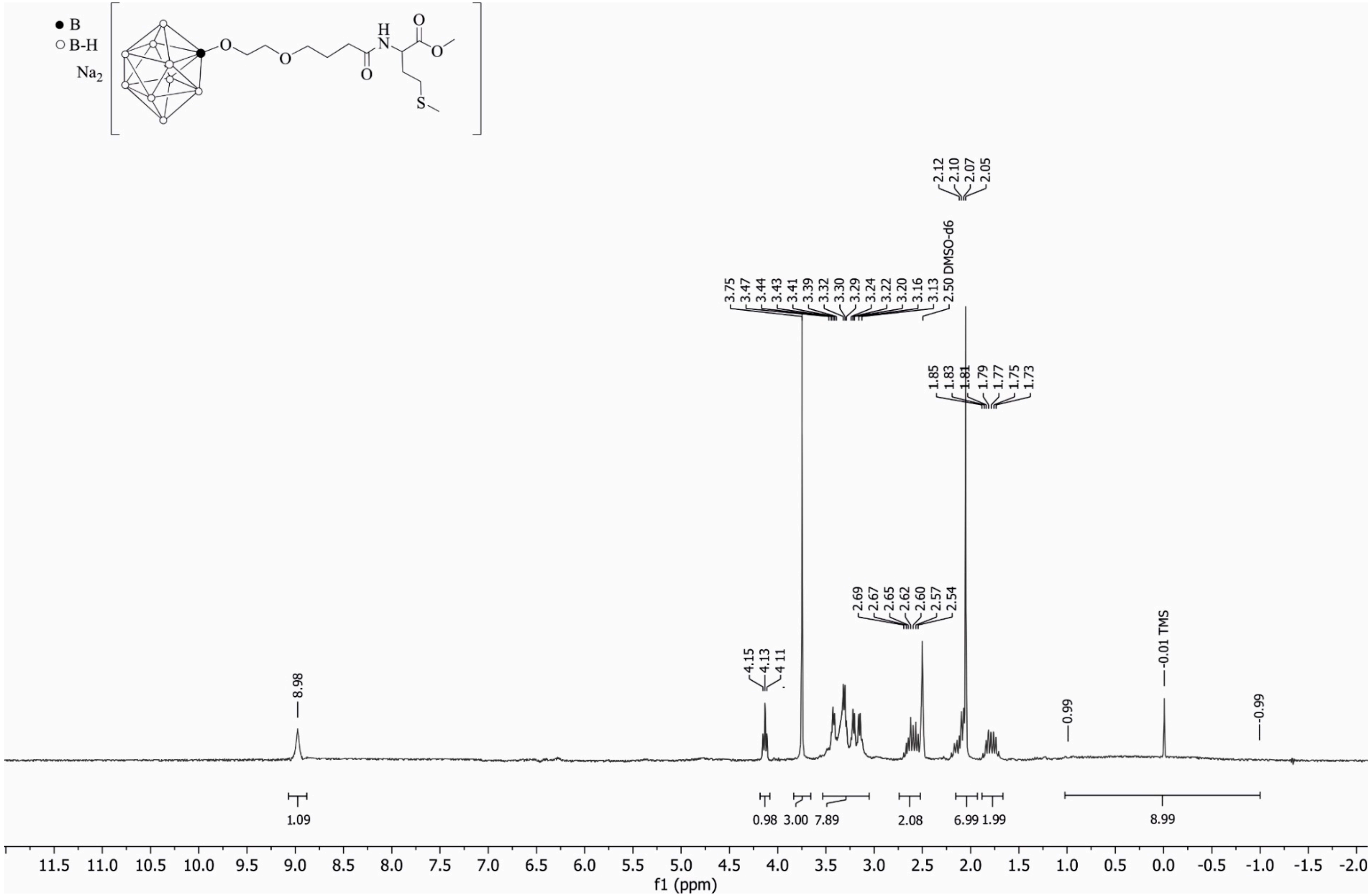

^{1}H NMR of **Na$_2$3**

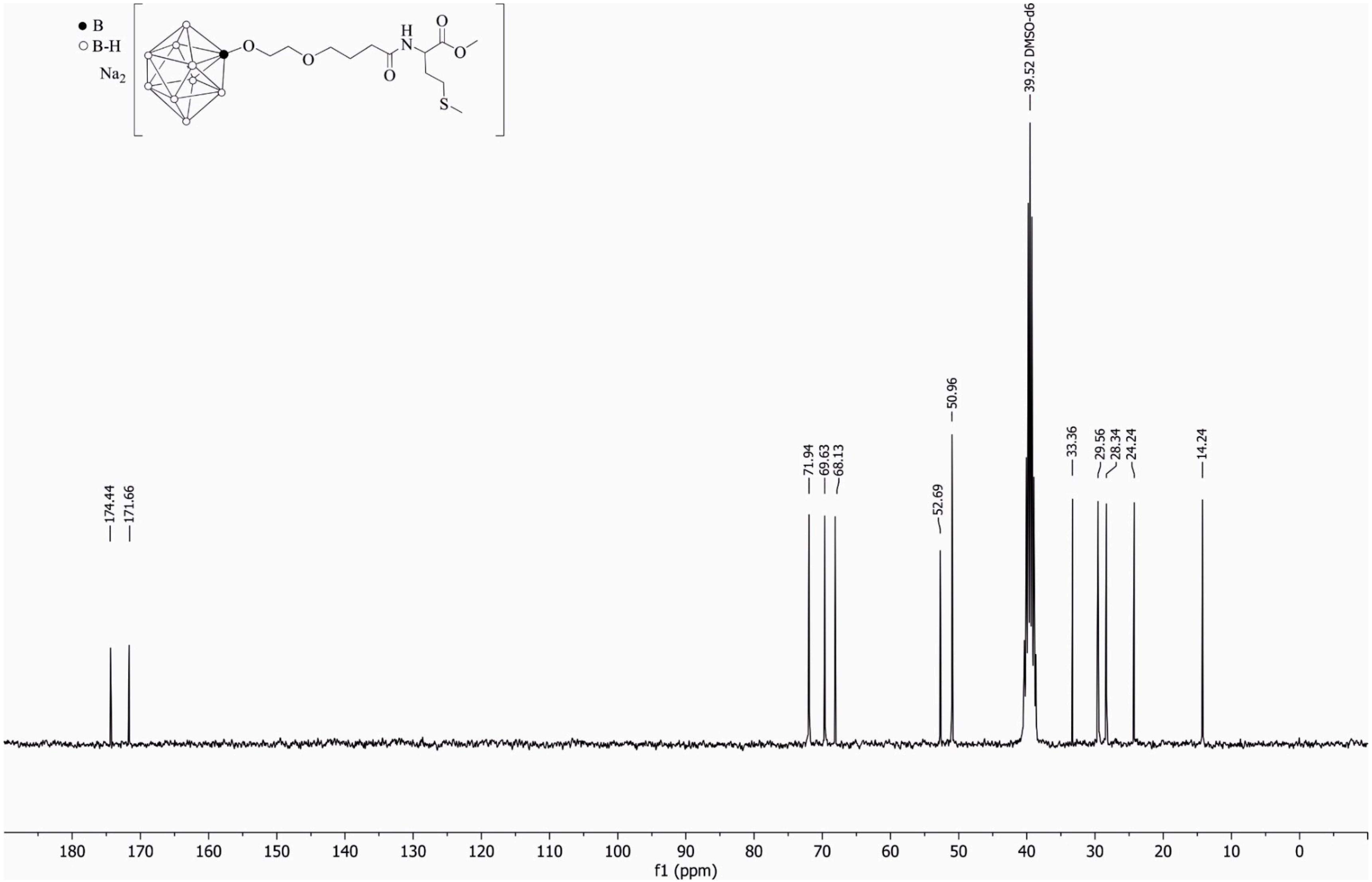

$^{13}C\{^{1}H\}$ NMR of **$Na_2$3**

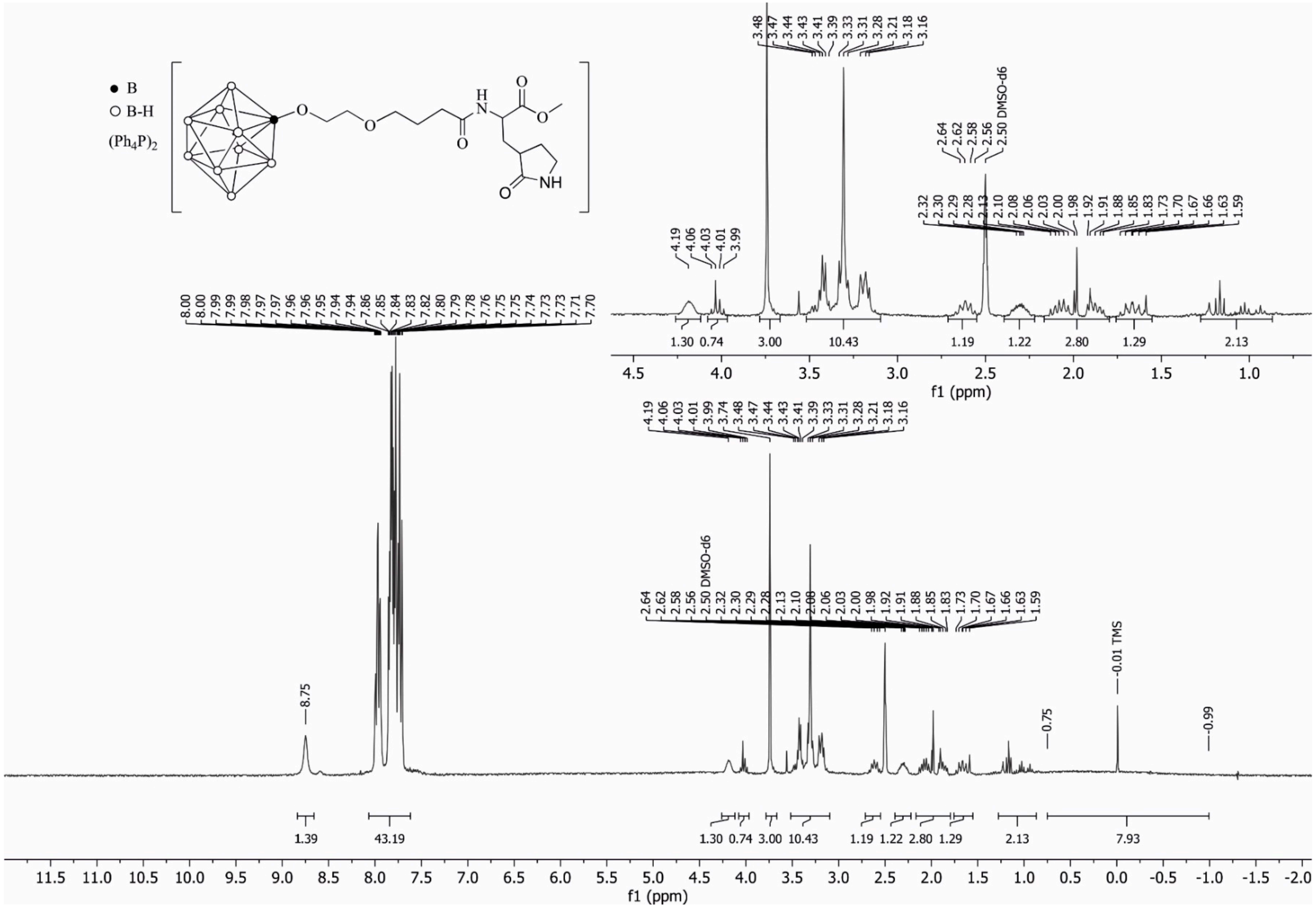

^{1}H NMR of **$(Ph_4P)_2$4**

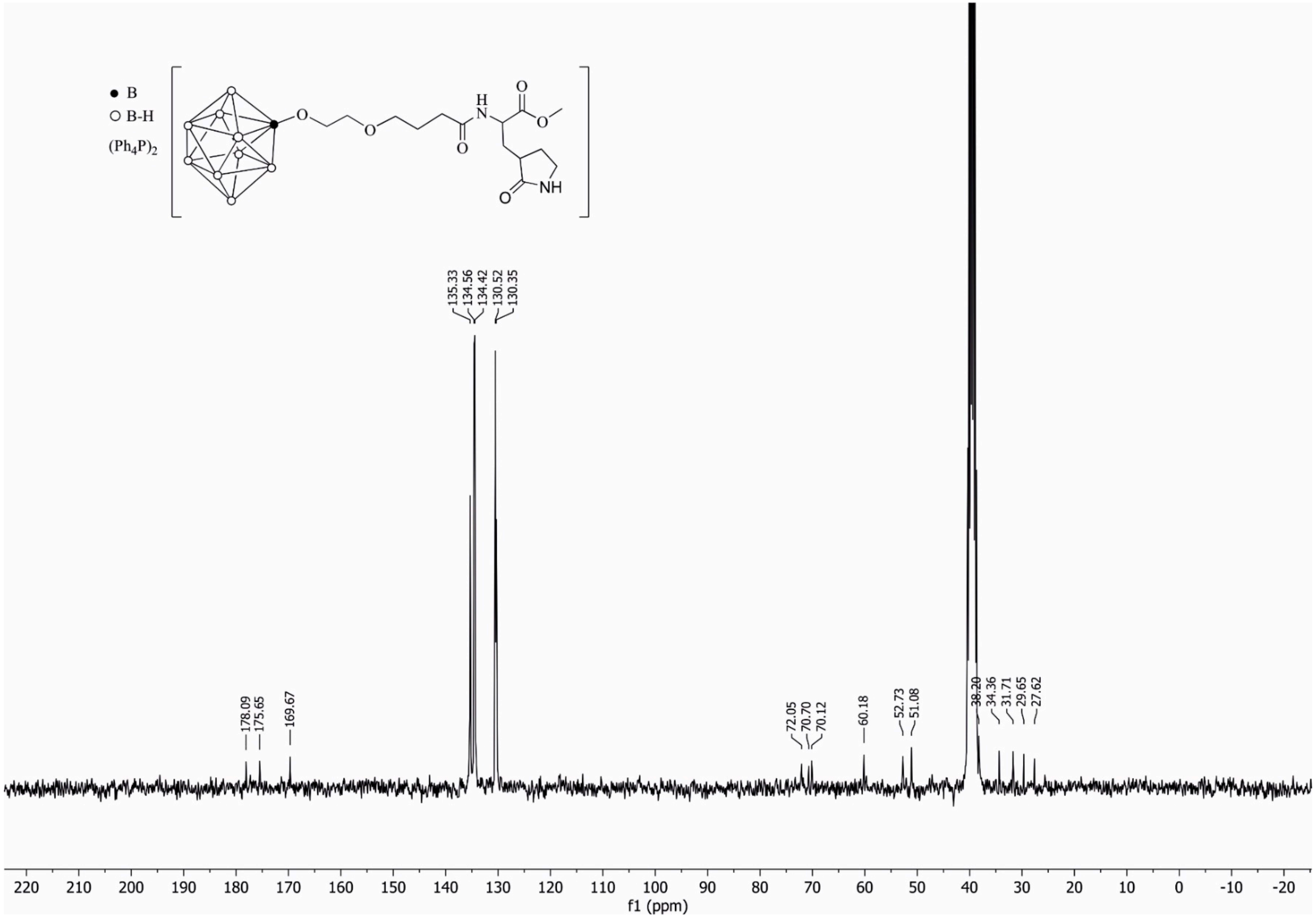

$^{13}C\{^1H\}$ NMR of **$(Ph_4P)_2$4**

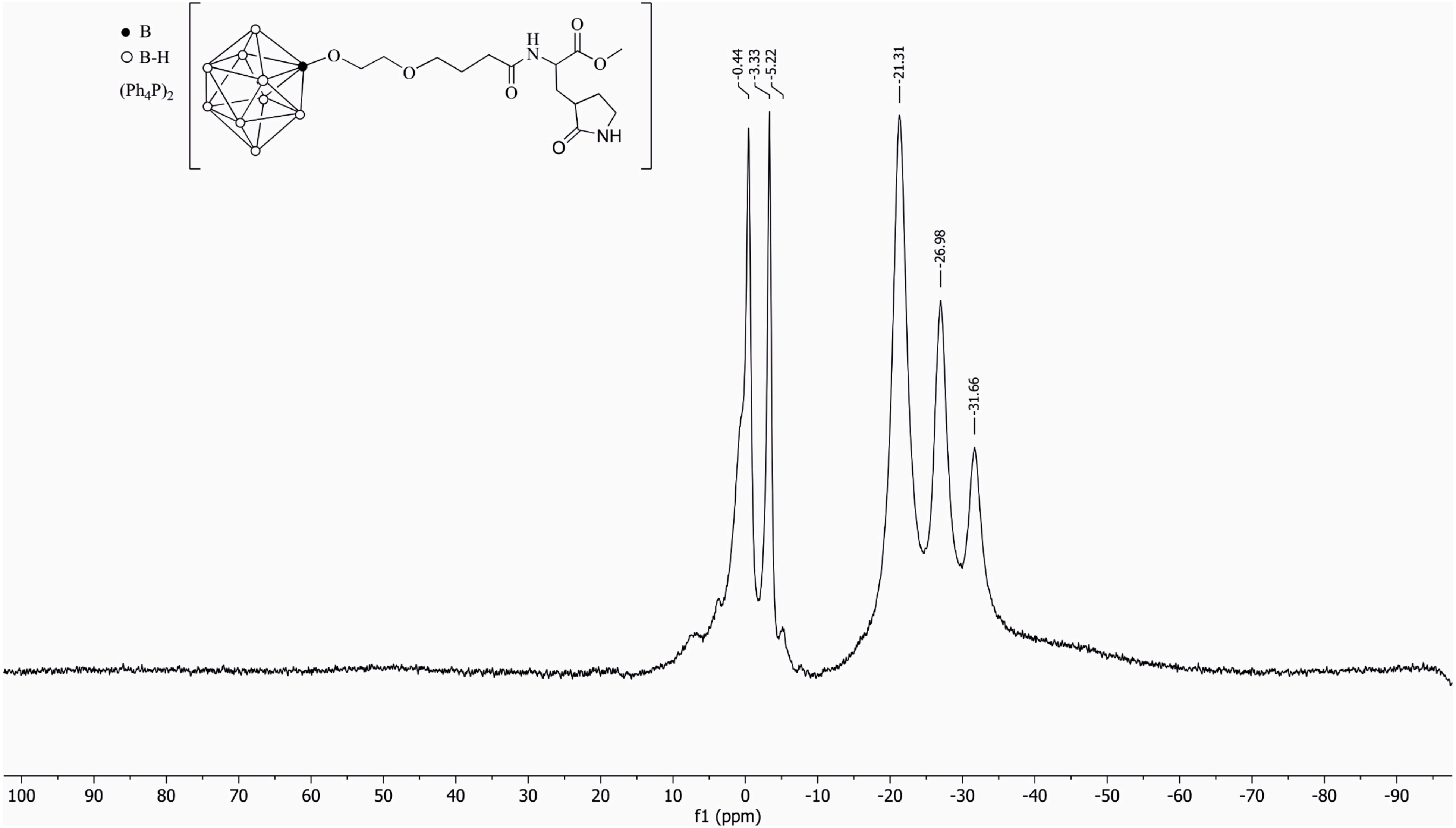

$^{13}B\{^{1}H\}$ NMR of **$(Ph_4P)_2$4**

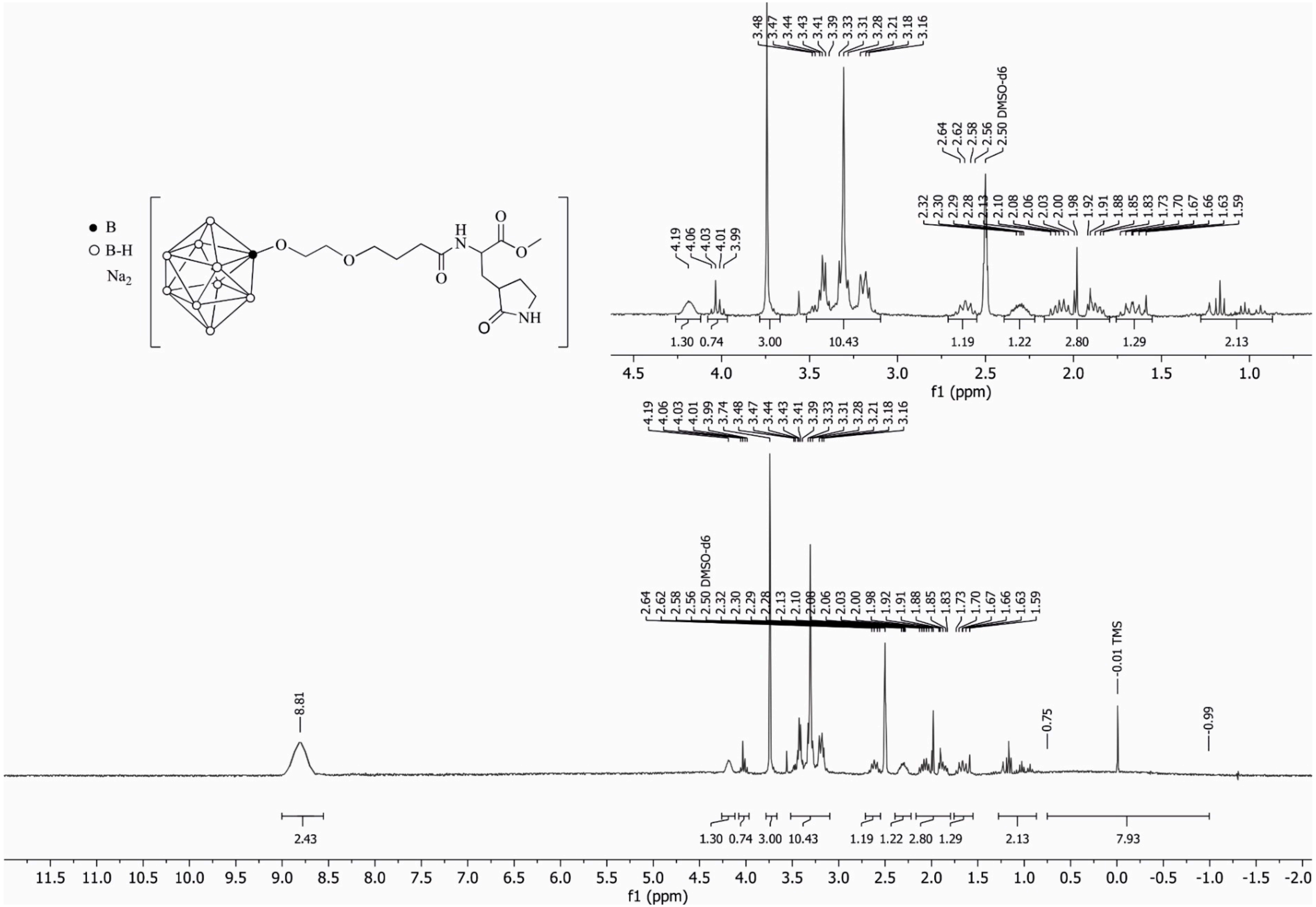

^{1}H NMR of **Na₂4**

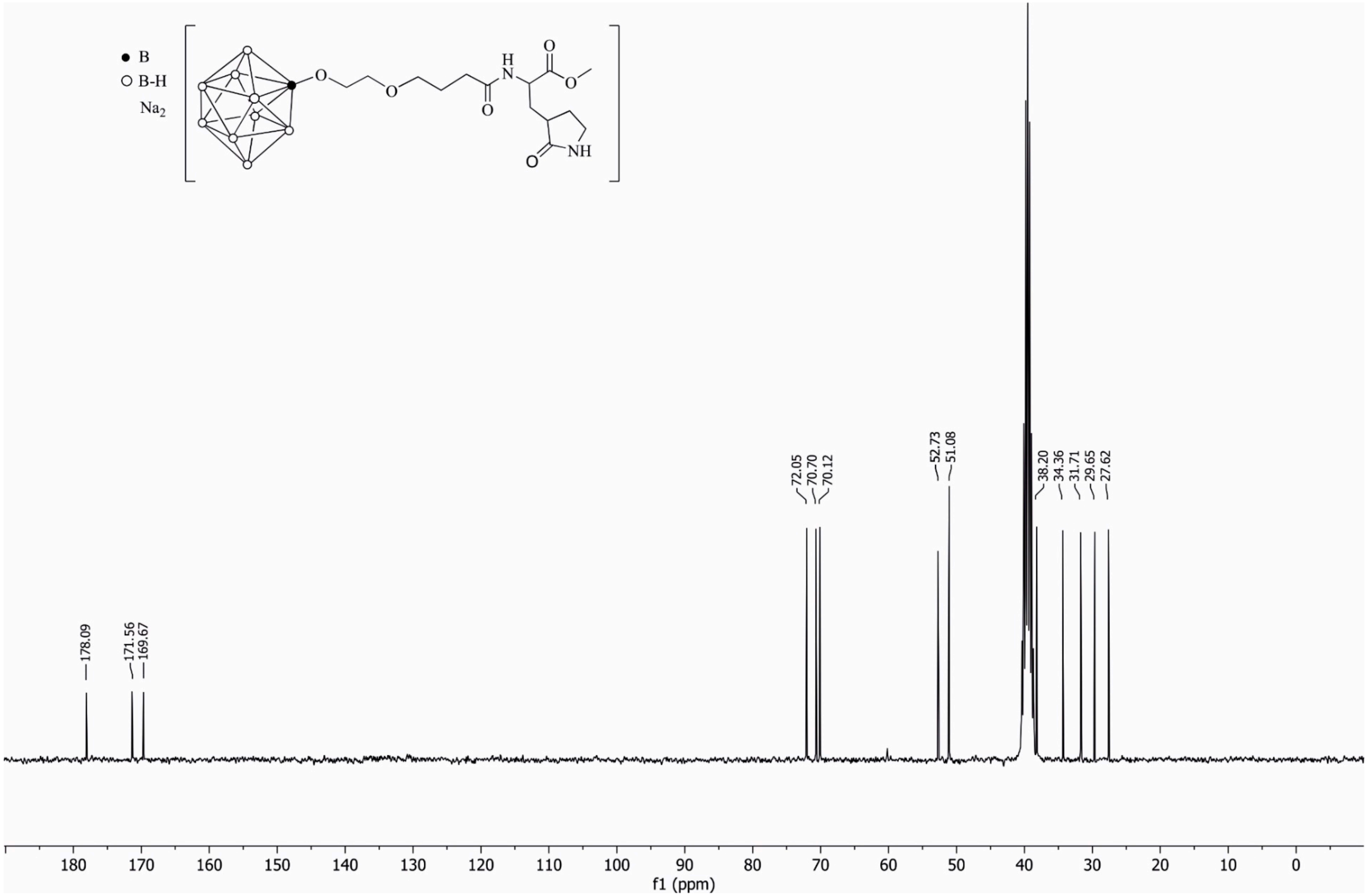

$^{13}C\{^{1}H\}$ NMR of **Na$_2$4**

IR spectroscopy data

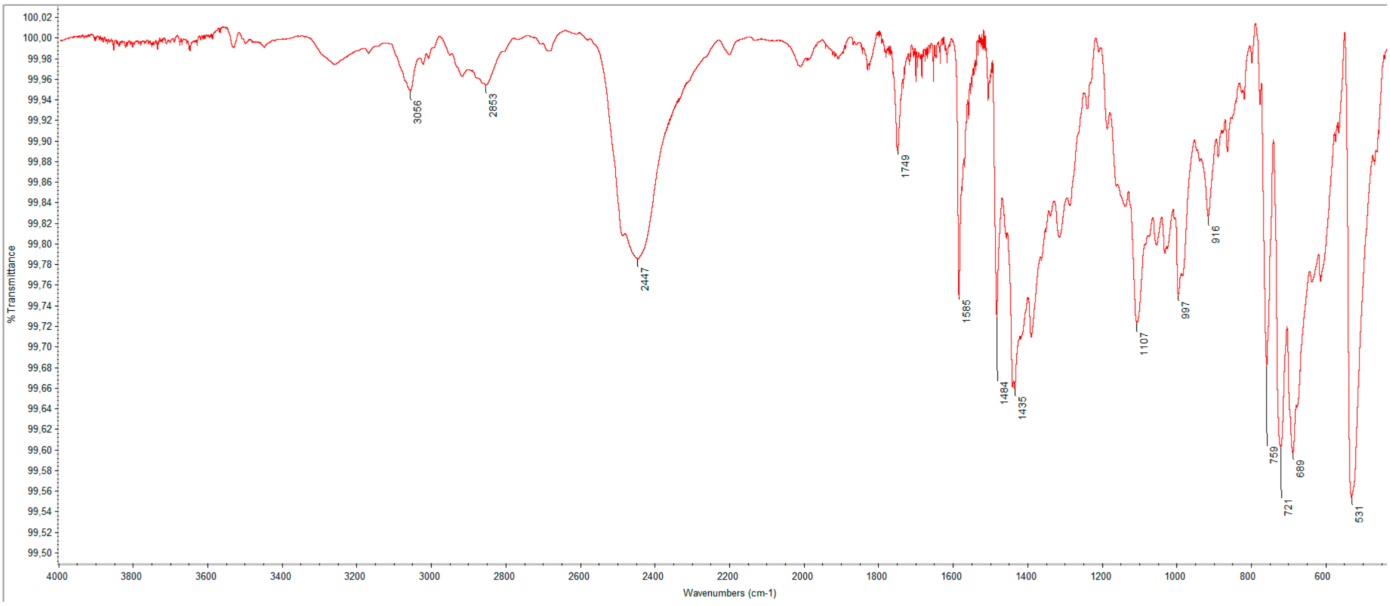

IR-spectrum $(Ph_4P)_2$**1** (KBr)

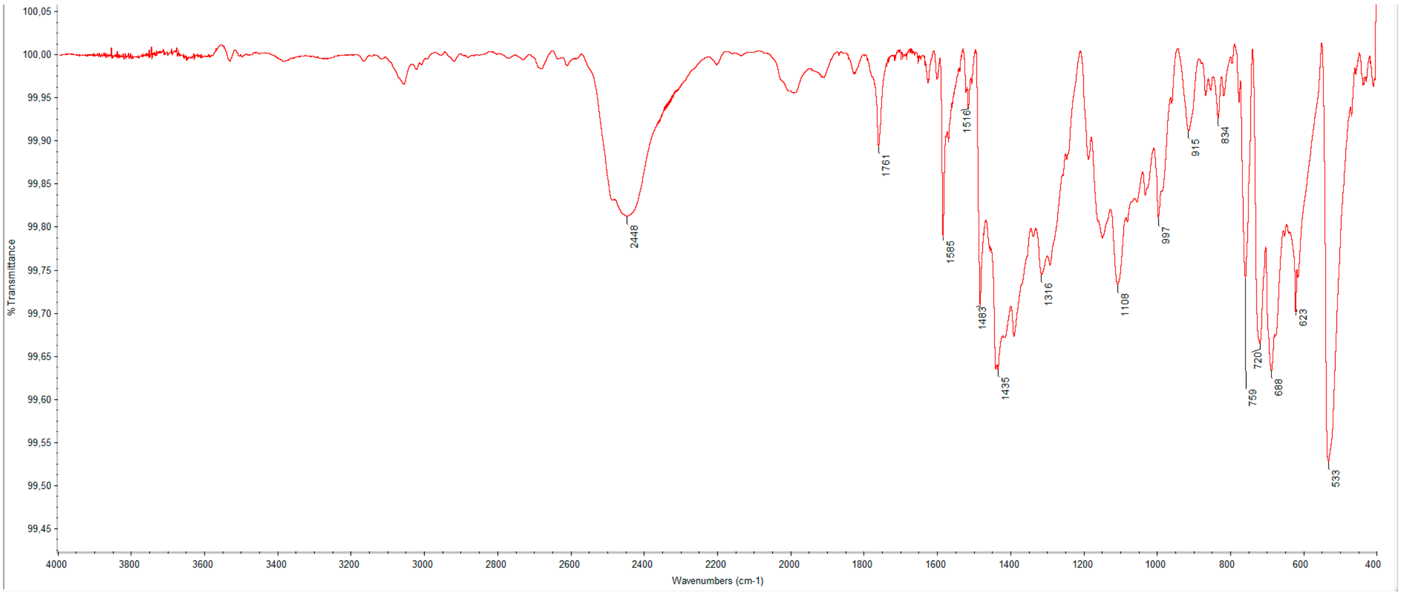

IR-spectrum of $(Ph_4P)_2$**2** (KBr)

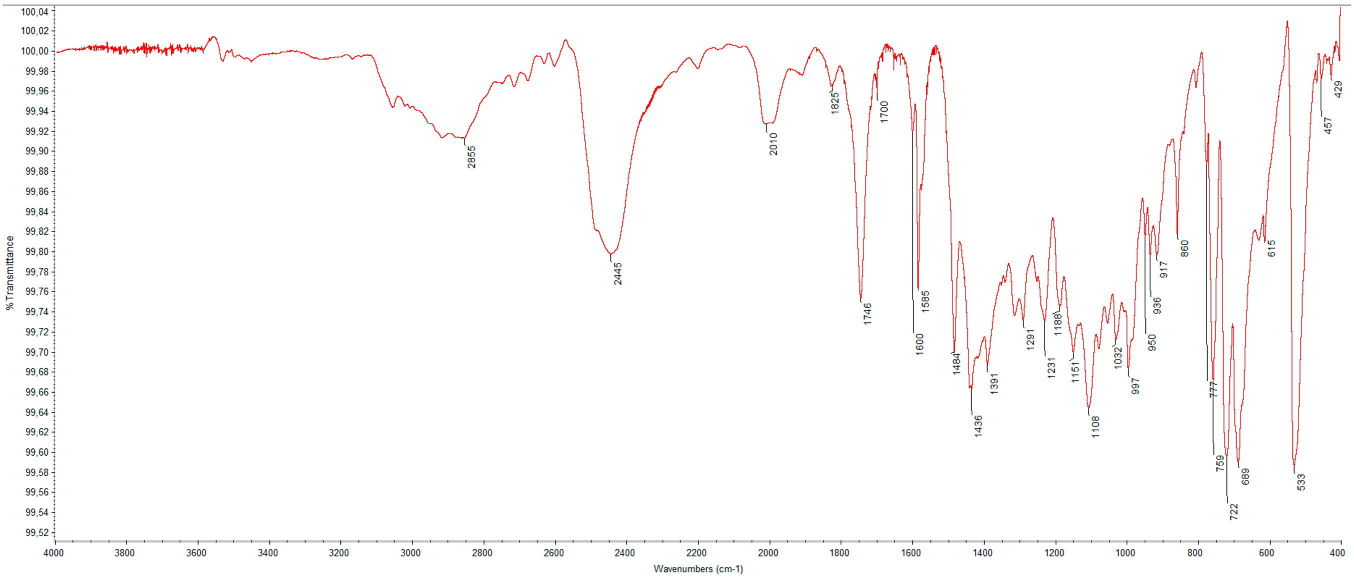

IR-spectrum of $(Ph_4P)_2$**3** (KBr)

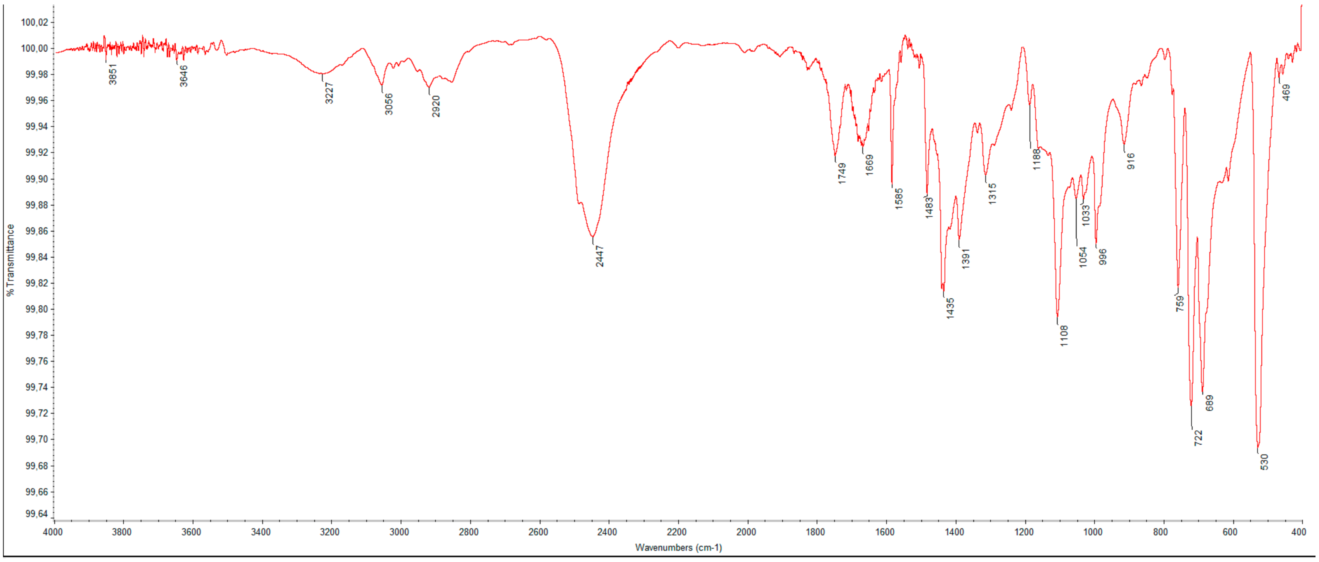

IR-spectrum of $(Ph_4P)_2$**4** (KBr)
